# Mapping transcription factor occupancy using minimal numbers of cells in vitro and in vivo

**DOI:** 10.1101/gr.227124.117

**Published:** 2018-04

**Authors:** Luca Tosti, James Ashmore, Boon Siang Nicholas Tan, Benedetta Carbone, Tapan K. Mistri, Valerie Wilson, Simon R. Tomlinson, Keisuke Kaji

**Affiliations:** 1 MRC Centre for Regenerative Medicine, University of Edinburgh, Edinburgh BioQuarter, Edinburgh, EH16 4UU, Scotland, United Kingdom

## Abstract

The identification of transcription factor (TF) binding sites in the genome is critical to understanding gene regulatory networks (GRNs). While ChIP-seq is commonly used to identify TF targets, it requires specific ChIP-grade antibodies and high cell numbers, often limiting its applicability. DNA adenine methyltransferase identification (DamID), developed and widely used in *Drosophila*, is a distinct technology to investigate protein–DNA interactions. Unlike ChIP-seq, it does not require antibodies, precipitation steps, or chemical protein–DNA crosslinking, but to date it has been seldom used in mammalian cells due to technical limitations. Here we describe an optimized DamID method coupled with next-generation sequencing (DamID-seq) in mouse cells and demonstrate the identification of the binding sites of two TFs, POU5F1 (also known as OCT4) and SOX2, in as few as 1000 embryonic stem cells (ESCs) and neural stem cells (NSCs), respectively. Furthermore, we have applied this technique in vivo for the first time in mammals. POU5F1 DamID-seq in the gastrulating mouse embryo at 7.5 d post coitum (dpc) successfully identified multiple POU5F1 binding sites proximal to genes involved in embryo development, neural tube formation, and mesoderm-cardiac tissue development, consistent with the pivotal role of this TF in post-implantation embryo. This technology paves the way to unprecedented investigation of TF–DNA interactions and GRNs in specific cell types of limited availability in mammals, including in vivo samples.

Genome-wide transcription factor (TF) occupancy is commonly assessed by chromatin immunoprecipitation followed by next-generation sequencing (ChIP-seq), but the need for both ChIP-grade antibodies and high numbers of cells limits its use (Barski et al. 2007). Although recent improvements of the ChIP-seq protocol, such as the iChIP and the ChIPmentation strategies (Lara-Astiaso et al. 2014; Schmidl et al. 2015), enabled the detection of histone marks across the genome using only 500 cells, they still require 10,000–500,000 cells to identify targets of the TF SPI1 (also known as PU.1). The use of CETCh-seq (CRISPR epitope tagging followed by ChIP-seq) overcomes the need for TF-specific ChIP-grade antibodies, but it does not reduce the required numbers of cells (Savic et al. 2015). DNA adenine methyltransferase identification (DamID) is a distinct method to investigate protein–DNA interactions, based on the exogenous expression of a protein of interest (POI) tethered to the DNA adenine methyltransferase (Dam) from *Escherichia coli* (van Steensel et al. 2001). Dam specifically methylates the adenine within a GATC sequence; hence, the expression of the Dam-POI fusion protein creates unique GA^me^TC marks on DNA adjacent to the POI binding sites. Subsequent genomic DNA (gDNA) extraction, digestion with the GA^me^TC-specific restriction enzyme DpnI, adapter ligation, PCR amplification and microarray (DamID-chip), or next-generation sequencing (DamID-seq) allow identification of the POI binding events. Unlike ChIP-seq, this technique does not require formaldehyde fixation or immunoprecipitation steps that could lead to data biases (Baranello et al. 2016) or loss of materials. DamID has been used in seminal studies in *Drosophila* for over 100 chromatin proteins and TFs (Moorman et al. 2006; Filion et al. 2010; van Bemmel et al. 2013). However, only limited success has been reported in mammalian cells due to technical difficulties. In particular, very low expression of the Dam protein without tethering POI (Dam-only) is sufficient to methylate DNA (Wines et al. 1996) since Dam itself can bind DNA and has highly processive methylation activity (Urig et al. 2002). The detection of POI-specific binding sites in DamID depends on the comparison of methylation signatures between Dam-only and Dam-POI–expressing cells. Thus, expressing Dam-only and Dam-POI at equally low levels in two independent populations is critical to identify POI-dependent methylation signals. This issue becomes even more relevant when the POI interacts with DNA at open chromatin loci (such as TFs), since Dam-only also preferentially binds and methylates nucleosome-free DNA (van Steensel et al. 2001). Since the first mammalian CBX1 DamID-chip paper (Vogel et al. 2006), only a handful of publications have reported the use of DamID-chip/seq for TFs in mammalian cells (Supplemental Table S1). We have overcome the aforementioned difficulties by applying translation reinitiation–mediated DamID, recently reported in *Drosophila* (Southall et al. 2013), to a mouse system. In combination with Tn5 transposase–mediated tagmentation and next-generation sequencing, this novel DamID-seq enabled us to detect clear TF binding signatures with as little as 1000 cells. This work details the improvements of the DamID-seq technology and demonstrates for the first time the identification of in vivo POU5F1 binding sites in the gastrulation-stage mouse embryo.

## Results

### Development of a translation reinitiation–mediated DamID-seq in mouse cells

In the original protocol for mammalian DamID-chip, Dam-only and Dam-CBX1 (formerly HP1β) were expressed via plasmid transfection under the ecdysone-inducible (Ec) promoter (Vogel et al. 2006). The leakiness of this promoter (i.e., in the absence of ecdysone) was sufficient to achieve an optimal, low expression of Dam-only/POI, and this strategy has been used for many DamID experiments in *Drosophila* Kc cells (Filion et al. 2010; van Bemmel et al. 2013). However, this approach limits the applicability of DamID where efficient transfection/viral infection or propagation of transfected cells is possible. In addition, expression levels of Dam-only/POI depend on transfection/infection efficiency, integration copy numbers, and integration sites; hence, achieving an equal expression level in two independent samples is technically challenging. Recently, the phenomenon of translation reinitiation has been exploited in *Drosophila* to achieve an optimal Dam expression level in a tissue-specific manner in combination with the GAL4-UAS system (Southall et al. 2013). Translation reinitiation takes place as the eukaryotic ribosome does not always detach from mRNA at the stop codon of a first open reading frame (ORF) and can restart translation of a second downstream ORF. Expression level of the protein encoded by the second ORF decreases as the length of the first ORF increases (Kozak 2001), providing a method by which to fine tune the level of Dam-only/POI expression (Southall et al. 2013).

To optimize translation reinitiation–mediated DamID in mammalian systems, we initially focused on the binding of the master regulator of pluripotency, POU5F1, in mouse embryonic stem cells (ESCs). Preceding DamID experiments, the functionality of the Dam-POU5F1 fusion protein was confirmed by maintaining an undifferentiated state in an inducible *Pou5f1* knockout ESC line (Supplemental Fig. S1).

**Figure 1.**
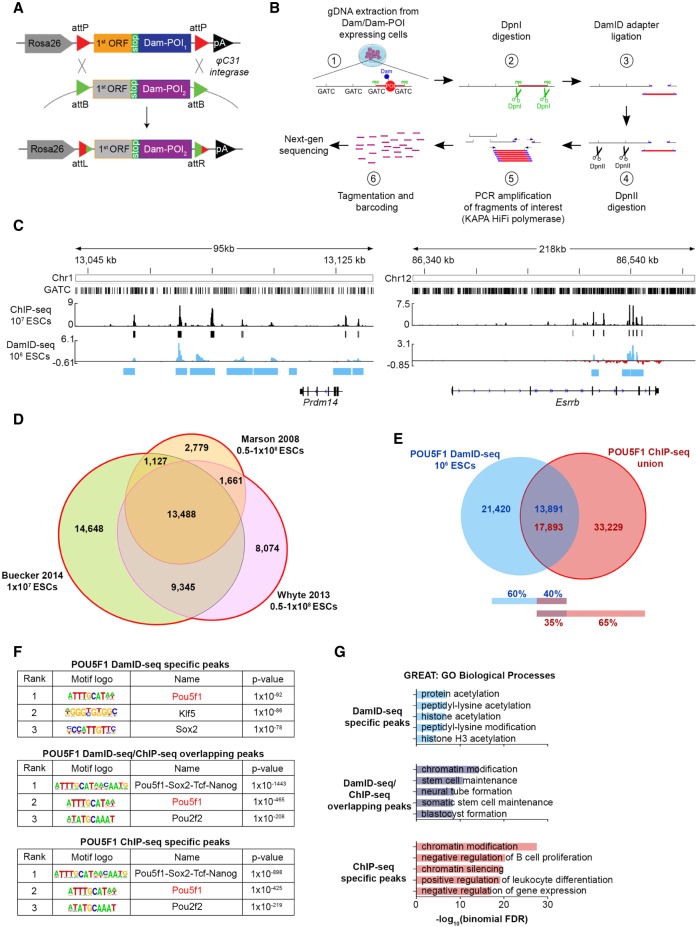
Optimization of DamID-seq in mouse embryonic stem cells (ESCs) and comparison with ChIP-seq. (*A*) φC31 integrase–mediated cassette exchange system used to generate the cell lines in this study. (POI) Protein of interest; (ORF) open reading frame. (*B*) The optimized DamID-seq workflow. (*C*) POU5F1 DamID-seq tracks generated from 10^6^ ESCs and POU5F1 ChIP-seq tracks generated from 10^7^ ESCs (Buecker et al. 2014). The bars *below* each track represent ChIP-seq (black) and DamID-seq (blue) statistically significant peaks; *y*-axis represents read counts per million of Dam-POU5F1 (Dam-subtracted) and POU5F1 ChIP-seq (input-subtracted), respectively. (*D*) Peak overlap between three different published POU5F1 ChIP-seq data sets (Marson et al. 2008; Whyte et al. 2013; Buecker et al. 2014)*.* (*E*) Overlap between POU5F1 DamID-seq peaks and the union of ChIP-seq peaks. Since DamID-seq peaks are larger than ChIP-seq peaks (see Supplemental Fig. S6B) and can contain multiple ChIP-seq peaks within, the number of the overlapping peaks are shown separately for each technology. (*F*) Motif enrichment analysis (Heinz et al. 2010) of the POU5F1-bound peaks identified only by DamID-seq, ChIP-seq, or both technologies. (*G*) Gene ontology (GO) enrichment analysis of POU5F1 peaks identified only by DamID-seq, ChIP-seq, or both using GREAT (McLean et al. 2010).

We then generated an ESC line containing a PhiC31 integrase–mediated cassette exchange platform within the *Gt(ROSA)26Sor* locus (Fig. 1A), allowing us to generate various cell lines with Dam-only/POI expression under the endogenous *Gt(ROSA)26Sor* promoter with high (∼100%) efficiency via simple plasmid transfection and drug selection.

To identify the optimal length of the first ORF for translation reinitiation–mediated DamID, we placed the *dam*-only/*dam-Pou5f1* coding sequences downstream from the stop codon of three different ORFs: blasticidin- (*Bsd*, 393 bp), neomycin- (*Neo*, 804 bp), and hygromycin- (*Hyg*, 1032 bp) resistance cassettes (Supplemental Fig. S2). Then we measured the methylation level at the *Pou5f1* locus in each cell line by quantitative PCR (qPCR)–based DamID (qDamID) (Supplemental Fig. S2; Methods).

When the *Bsd* cassette was used, the Dam-only methylation level at many of the tested DNA loci was >70%, indicating that Dam expression was too high, although POU5F1 binding was observed at the previously described target site O4 (Supplemental Fig. S2A). The use of *Neo* cassette successfully decreased the methylation levels, generating a POU5F1 binding signature similar to ChIP-seq data (Supplemental Fig. S2A). The longer ORF *Hyg* cassette made the methylation signals even lower and the overall signals indistinguishable between Dam-only and Dam-POU5F1–expressing cell lines. Thus, we opted to use the *Neo* cassette as the first ORF, and these cell lines were referred to as *Rosa26-Neo-Dam* and *Rosa26-Neo-Dam-Pou5f1* ESC lines in the subsequent experiments.

### Translation reinitiation–mediated DamID enables the detection of TF binding dynamics during ESC differentiation

One of the big advantages of generating Dam-only/POI–expressing ESC lines is that they can be differentiated into any cell type. The *Rosa26-Neo-Dam* and *Rosa26-Neo-Dam-Pou5f1* ESC lines differentiated similarly well into epiblast-like cells (EpiLCs) (Supplemental Fig. S3A) and into fibroblast-like cells in vitro. Since the protein level of Dam-only/POI via translation reinitiation is extremely low, it is unlikely to affect the functionality of the cells. *Neo-Dam* and *Neo-Dam-Pou5f1* mRNA levels remained constant after differentiation as we used the ubiquitously active endogenous *Gt(ROSA)26Sor* promoter (Supplemental Fig. S3A,B). In contrast, when we placed the Ec promoter-driven *Dam-only/Pou5f1* cassettes in the *Gt(ROSA)26Sor* locus, the transgenes were silenced following differentiation into fibroblast-like cells (Supplemental Fig. S3C). Although the *Rosa26-Neo-Dam* and *Rosa26-Neo-Dam-Pou5f1* lines constitutively express Dam-only/Dam-POU5F1, we observed loss and gain of the POU5F1 binding signatures using a 72-h EpiLC differentiation protocol (Supplemental Fig. S3D), in agreement with ChIP-seq data. In summary, these results indicated that the translation reinitiation–based DamID system could detect distinct POI binding signal in different cell types, as long as cells divide and dilute the methylation signal generated in previous differentiation stages.

### Optimization of DamID-seq protocol

A few studies have described the use of DamID-seq for the analysis of TF binding in mammalian cells (Supplemental Table S1), yet with little modifications of the original DamID-chip protocol (Vogel et al. 2007). Thus, we revisited each step of the protocol and optimized it for DamID-seq (Fig. 1B). gDNA was extracted using the Quick-gDNA MicroPrep kit (Zymo Research) and then digested with DpnI, which specifically cuts GA^me^TC sequences. Following adapter ligation, DNA was digested with DpnII (which specifically cuts nonmethylated GATC sites) before adapter-mediated PCR amplification. The DpnII digestion step was described in the original protocol to avoid amplification of large fragments that do not contain Dam/Dam-POI–bound sites but are flanked by the DpnI-digested GA^me^TC sites. Although this step has been excluded in recent protocols (Bouveret et al. 2015; Jacinto et al. 2015; Kind et al. 2015), we found that the intensity of the Dam-POU5F1 signal over the Dam-only control was reduced without this DpnII digestion step (Supplemental Fig. S4A). POU5F1 DamID has been recently used to validate a DamID-seq protocol in a different study (Jacinto et al. 2015). The data from Jacinto et al. (2015) presented lower signal-to-noise ratio compared to our data, maybe due to the lack of DpnII digestion step before PCR amplification in their protocol and/or potentially due to the difficulty in achieving optimal expression levels of *Dam*-only/*Pou5f1* using viral transduction (Supplemental Fig. S5).

For the amplification of the adapter-ligated DNA fragments, we found that the KAPA HiFi polymerase provided a better genome coverage than Advantage2 polymerase previously used (Supplemental Fig. S4B; van Steensel et al. 2001; Guelen et al. 2008; Southall et al. 2013). We also introduced a qPCR step to determine the optimal number of PCR cycles for the fragment amplification in order to minimize amplification biases (Supplemental Fig. S4C). All these steps could be performed in a single tube, and the amplified DNA was then purified using SPRI magnetic beads. The purified Dam-only/POI target DNA was subjected to library preparation for Illumina sequencing with Tn5 transposition (Picelli et al. 2014), which allowed us to fragment the DNA to the desired size range (∼250–350 bp) for NGS and to introduce Illumina sequencing–compatible ends in a 5-min reaction. This DamID-seq protocol (from gDNA extraction to NGS library preparation) can be accomplished in ∼3 d.

### POU5F1 DamID-seq with 10^6^ ESCs and comparison with ChIP-seq

We initially performed POU5F1 DamID-seq using 10^6^ ESCs. Visual inspection of the POU5F1 DamID-seq tracks, generated by subtraction of the Dam-only signal from the Dam-POU5F1 signal, revealed good agreement with POU5F1 ChIP-seq data generated with 10^7^ ESCs (Fig. 1C). We reanalyzed three previously published POU5F1 ChIP-seq data sets (Fig. 1D; Marson et al. 2008; Whyte et al. 2013; Buecker et al. 2014) and used the union of the identified peaks (51,122 in total) for further comparison with the 35,311 POU5F1 DamID-seq peaks identified in this study using a new DamID-seq data analysis pipeline described in the Methods (the source code is available in Supplemental Material S1). About 40% of the POU5F1 DamID-seq peaks overlapped with 35% of the POU5F1 ChIP-seq peaks (Fig. 1E). This overlap was larger than that between standard formaldehyde-crosslinked ChIP-seq and recently published UV laser–crosslinked ChIP-seq using the same antibody (Steube et al. 2017). Motif enrichment analysis confirmed the enrichment of the POU5F1 motif in the overlapping peaks, as well as in DamID-specific and in ChIP-specific peaks (Fig. 1F). Overall, a substantial number of peaks contained the POU5F1 motif, specifically: ∼67% of the DamID-seq peaks and ∼30% of the ChIP-seq peaks (Supplemental Fig. S6A,B). However, both peak size and location of the motif within each peak were distinct between DamID-seq and ChIP-seq (Supplemental Fig. S6C,D). The mode of DamID-seq peak size was 1143 bp (Supplemental Fig. S6C), and the distribution of POU5F1 motif was widely spread across ±2 kb from the peak center (Supplemental Fig. S6D). The mode of ChIP-seq peak size was 334 bp with the POU5F1 motif in the center of the peak (Supplemental Fig. S6C,D). When we randomly selected the same numbers of genomic regions while maintaining the same size distribution as DamID-seq or ChIP-seq peaks, we found that ∼53% and ∼15% of these random regions contained the POU5F1 motif, respectively (Supplemental Fig. S6A,B). Hence, we concluded that the higher presence of the POU5F1 motif in DamID-seq peaks was due to their bigger size compared with ChIP-seq peaks and that both technologies can enrich for genomic regions containing the POU5F1 motif.

Interestingly, DamID-specific peaks were highly enriched with SOX2 and KLF5 motifs, instead of the POU5F1-SOX2-TCF-NANOG binding motif enriched in the overlapping and ChIP-seq–specific peaks (Fig. 1F). POU5F1 and SOX2 bind synergistically to the OCT/SOX motif with higher affinity (Ambrosetti et al. 1997; Mistri et al. 2015), while other pluripotency genes co-occupy their targets without such a known synergistic effect. Since DamID can detect transient or weak protein–DNA interaction (Aughey and Southall 2016), DamID-specific peaks may reflect the sensitive nature of this technology. When we performed Gene Ontology (GO) enrichment analysis using GREAT (McLean et al. 2010), GO terms associated with the overlapping peaks included “stem cell maintenance,” “blastocyst formation,” and “neural tube formation” (Fig. 1G) in agreement with known POU5F1 function in mESCs and in embryonic development (Niwa et al. 2000; Simandi et al. 2016). On the contrary, POU5F1 DamID-seq– and ChIP-seq–specific peaks were associated with GO terms not related to previously described POU5F1 functions.

**Figure 2.**
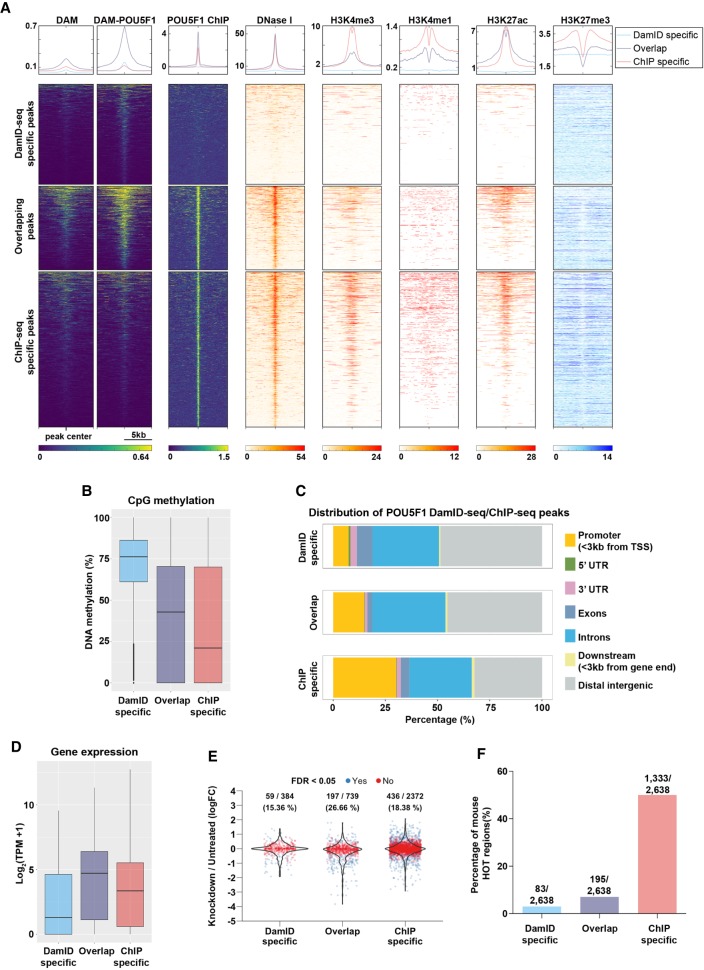
Characterization of POU5F1 DamID-seq and ChIP-seq peaks. (*A*) For each POU5F1 DamID/ChIP-seq overlapping and specific peaks, the signal intensities from POU5F1 DamID-seq (Dam; Dam-POU5F1 separately), POU5F1 ChIP-seq, DNase-seq, and H3K4me3, H3K4me1, H3K27Ac, and H3K27me3 ChIP-seq are represented. (*B*) CpG methylation levels of POU5F1 DamID/ChIP-seq overlapping and specific peaks. The ChIP-seq–specific peaks were extended to the same average size of DamID-seq peaks as to avoid biases due to different size of the peaks. (*C*) Distribution of POU5F1 DamID/ChIP-seq overlapping and specific peaks in the genome. (*D*) Expression level of genes with DamID-seq–specific (384 genes), overlapping (739 genes), and ChIP-seq–specific peaks (2372 genes) in their promoter regions (±3 kb from TSS). (*E*) Expression changes of genes in *D*, 24 h after knockdown of *Pou5f1* in ESCs (King and Klose 2017). (*F*) DamID/ChIP-seq overlapping and specific peaks including mouse ChIP-seq HOT regions (Wreczycka et al. 2017).

When we investigated the features of DamID/ChIP-seq–specific peaks, we found that ChIP-specific peaks were nucleosome-free (measured by DNase-seq), similar to the overlapping peaks, but they showed higher levels of H3K4me3 and, to a lesser extent, H3K27me3 and H3K4me1 (Fig. 2A). Conversely, DamID-seq–specific peaks were not nucleosome-free, with very low Dam-only signal consistent with the fact that Dam-only preferentially binds to nucleosome-free DNA. Higher Dam-POU5F1 signal in those loci potentially reflects the pioneering activity of POU5F1 (Fig. 2A; Soufi et al. 2015). DamID-seq–specific peaks were not enriched with any of these active histone modifications or repressive H3K27me3 mark (Fig. 2A). Instead, they showed ∼75% CpG methylation, while the ChIP-seq–specific peaks had only ∼25% CpG methylation (Fig. 2B). Consistent with these features, only ∼10% of the DamID-specific peaks, but ∼30% of the ChIP-specific peaks were located in the gene promoters (Fig. 2C). The genes containing DamID-seq peaks in the promoter were expressed at lower levels than those containing ChIP-seq peaks (Fig. 2D). About 15% and 18% of those genes were up- or down-regulated (FDR < 0.05) 24 h after termination of *Pou5f1* expression (King and Klose 2017), while >26% of the genes with overlapping peaks in the promoter regions changed their expression (Fig. 2E). The features of ChIP-seq–specific peaks (enrichment of H3K4me3, close proximity to TSS, low CpG methylation, high expression of target genes) resembles the regions described as “hyper-ChIPable” or “phantom peaks” in yeast and in fly (Park et al. 2013; Teytelman et al. 2013; Jain et al. 2015) and, more recently, as “HOT regions” in worm, mouse, and human ChIP-seq data (Wreczycka et al. 2017). Notably, ∼51% of the mouse HOT regions overlapped with POU5F1 ChIP-seq–specific peaks, while only ∼3% of them overlapped with POU5F1 DamID-specific peaks (Fig. 2F). Since Dam-only tends to methylate open loci, the comparison between Dam-only and Dam-POU5F1 samples in DamID-seq is likely to exclude the HOT regions from the peak list, while some of the ChIP-seq–specific peaks could represent true POU5F1 binding sites not identified by DamID-seq.

### SOX2 DamID-seq with 10^6^ NSCs and comparison with ChIP-seq

**Figure 3.**
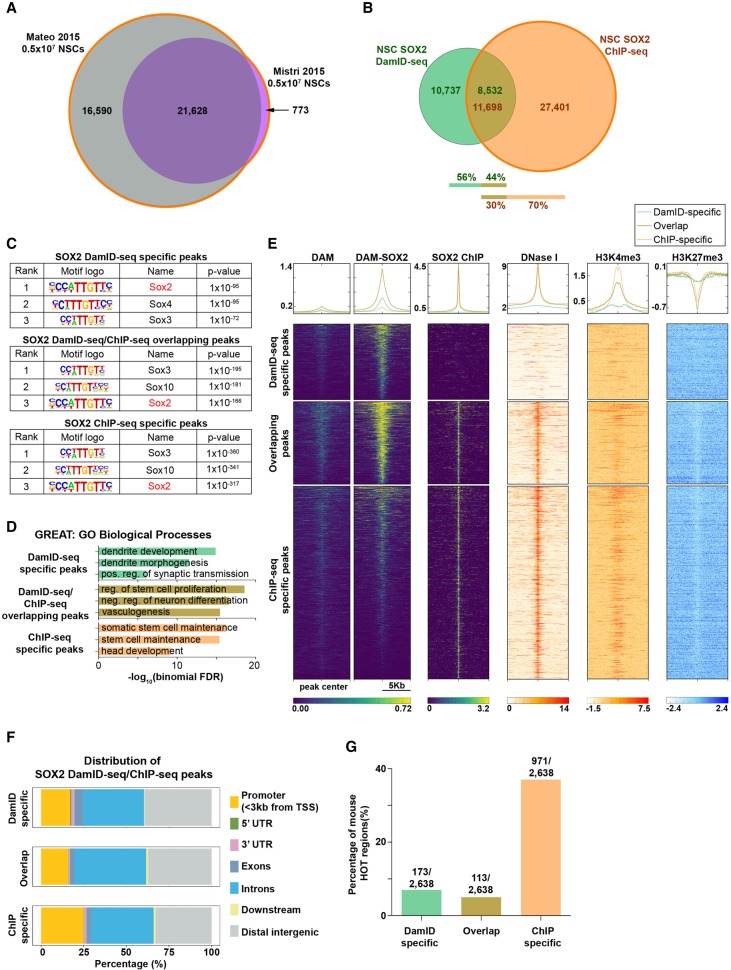
10^6^ NSC SOX2 DamID-seq in comparison with ChIP-seq. (*A*,*B*) Union of two published NSC SOX2 ChIP-seq data sets (*A*) (Mateo et al. 2015; Mistri et al. 2015) and its overlap with 10^6^ NSC SOX2 DamID-seq peaks (*B*). (*C*) Motif enrichment analysis (Heinz et al. 2010) of the SOX2-bound peaks identified only by DamID-seq, ChIP-seq, or both technologies. (*D*) GO enrichment analysis of SOX2 peaks identified only by DamID-seq, ChIP-seq, or both using GREAT (McLean et al. 2010). (*E*) For each SOX2 DamID/ChIP-seq overlapping and specific peaks, the signal intensities from SOX2 DamID-seq (Dam; Dam-SOX2 separately), SOX2 ChIP-seq, DNase-seq, and H3K4me3 and H3K27me3 ChIP-seq are represented. (*F*) Distribution of SOX2 DamID/ChIP-seq overlapping and specific peaks in the genome. (*G*) DamID/ChIP-seq overlapping and specific peaks including mouse ChIP-seq HOT regions (Wreczycka et al. 2017).

To confirm our observations about the features of DamID-seq data in different cell types and different TFs, we performed SOX2 DamID-seq in mouse NSCs differentiated in vitro from *Rosa26-Neo-Dam* and *Rosa26-Neo-Dam-Sox2* ESC lines. For the comparison with the 10^6^ NSC DamID-seq data, we used the union of two published NSC SOX2 ChIP-seq data (Fig. 3A; Mateo et al. 2015; Mistri et al. 2015). Similar to the ESC POU5F1 DamID-seq/ChIP-seq comparison, ∼44% of SOX2 NSC DamID-seq peaks overlapped with ∼30% of ChIP-seq peaks (Fig. 3B). These overlapping peaks as well as DamID-seq/ChIP-seq–specific peaks showed enrichment of the SOX2 motif and NSC-related GO terms following GREAT analysis (Fig. 3C,D). Percentages of peaks with the SOX2 motif, peak size distribution and motif position were similar to what we observed in POU5F1 DamID-seq and ChIP-seq in ESCs (Supplemental Fig. S7). Similarly, the SOX2 DamID-seq–specific peaks were also devoid of signals from DNase-seq and from H3K4me3 and H3K27me3 ChIP-seq, and the SOX2 ChIP-seq–specific peaks had stronger DNase-seq and H3K4me3 ChIP-seq signals (Fig. 3E). Furthermore, NSC SOX2 ChIP-seq–specific peaks also had the highest proportion (∼25%) of peaks in the promoter regions, including ∼37% of mouse HOT regions (Fig. 3F,G).

In summary, we have shown that TF DamID-seq with about 10-fold fewer cells (10^6^ cells) can identify POU5F1 and SOX2 targets in ESCs and NSCs, respectively, of which 40%–44% were also identified by ChIP-seq. Considering the fundamental differences between the two techniques, as well as the scarcity of strategies to validate ChIP-seq data, this reproducible overlap rate in two different TFs and cell types is encouraging. Importantly, the overlap between ESC POU5F1 DamID-seq and NSC SOX2 DamID-seq peaks was as small as that between ESC POU5F1 ChIP-seq and NSC SOX2 ChIP-seq peaks (Supplemental Fig. S8), indicating that the technique-dependent nonspecific peaks in DamID-seq are as few as those in ChIP-seq. A number of nonoverlapping peaks with distinct features exist in both techniques, and their functional importance needs to be carefully studied in future. Nevertheless, the overlapping peaks have stronger signals in both DamID-seq and ChIP-seq (Figs. 2A, 3E; Supplemental Fig. S9). Hence, a possible strategy to reduce the technique specific peaks and enrich peaks that can be identified by both DamID-seq and ChIP-seq would be increasing the threshold of the peak calling.

### TF DamID-seq in 1000 flow-sorted ESCs and NSCs

**Figure 4.**
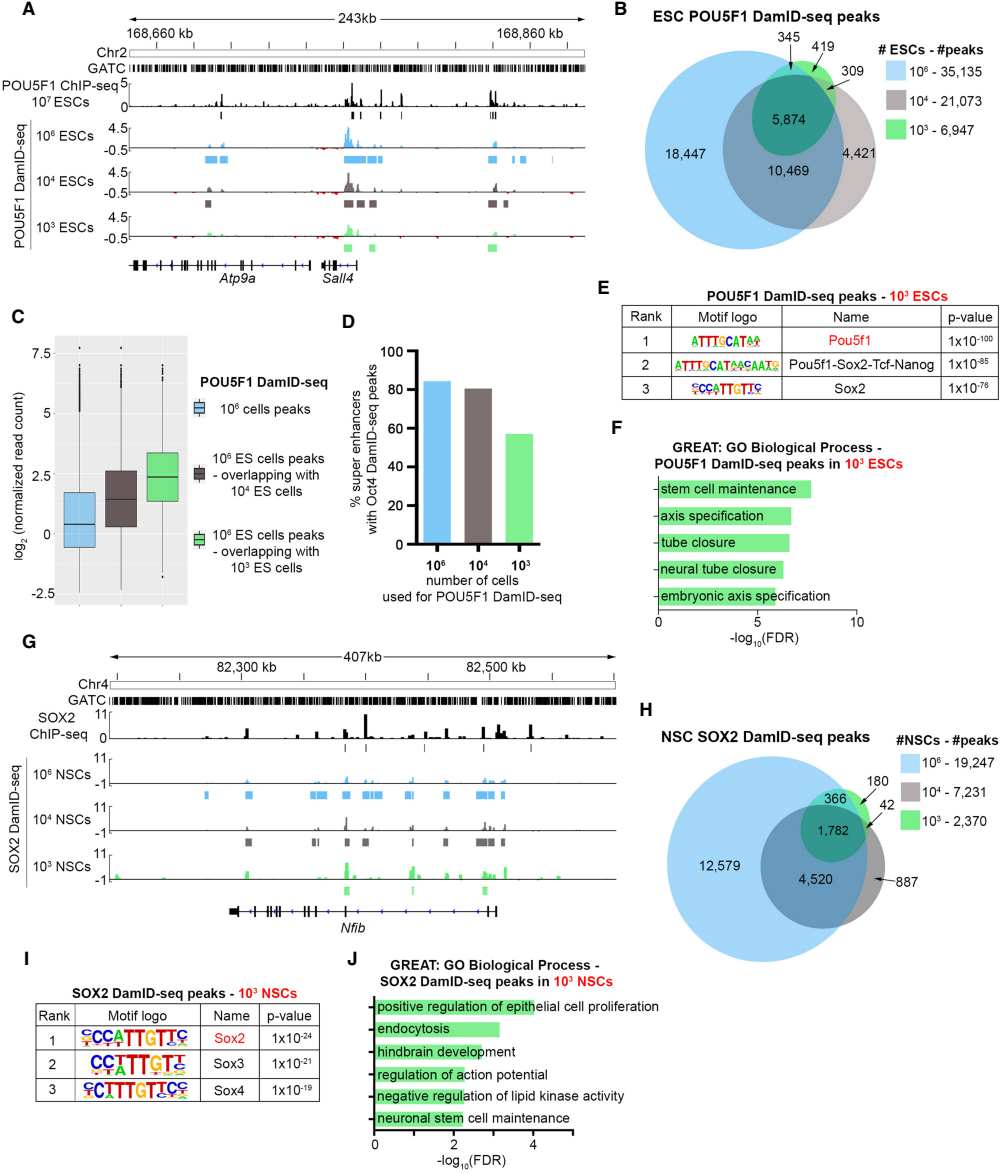
10^4^, 10^3^ ESC POU5F1 DamID-seq and 10^4^, 10^3^ NSC SOX2 DamID-seq. (*A*) POU5F1 DamID-seq tracks from 10^6^/10^4^/10^3^ ESCs and POU5F1 ChIP-seq track from 10^7^ ESCs (Buecker et al. 2014). (*B*) Overlaps of 10^6^/10^4^/10^3^ ESC POU5F1 DamID-seq peaks. (*C*) Read counts of peaks in 10^6^ ESC DamID-seq (blue) and those identified by 10^4^ (gray) and 10^3^ (green) ESC DamID-seq. (*D*) Percentage of ESC super enhancers (Whyte et al. 2013) containing POU5F1 DamID-seq peaks using different number of cells. (*E*) Motif enrichment in the 10^3^ ESC POU5F1 DamID-seq peaks. (*F*) GO enrichment analysis of 10^3^ ESC POU5F1 DamID-seq peaks using GREAT (McLean et al. 2010) (*G*) SOX2 DamID-seq tracks from 10^6^/10^4^/10^3^ NSCs and SOX2 ChIP-seq tracks generated from 5 × 10^6^ NSCs (Mateo et al. 2015). (*H*) Overlaps of 10^6^/10^4^/10^3^ NSC SOX2 DamID-seq peaks. (*I*) Motif enrichment in the 10^3^ NSC SOX2 DamID-seq peaks. (*J*) GO enrichment analysis of SOX2 DamID-seq peaks from 10^3^ NSCs.

Since the DamID protocol does not include any precipitation procedure, which causes a loss of DNA, we reasoned that it could be applied to lower numbers of cells. Accordingly, we performed POU5F1 DamID-seq with 10^4^ and 10^3^ *Rosa26-Neo-Dam* and *Rosa26-Neo-Dam-Pou5f1* ESCs (Fig. 4A). While the number of statistically significant POU5F1 peaks decreases when using fewer cells, the peaks highly overlap with those identified from 10^6^ ESCs (Fig. 4B). Peaks identified from 10^4^ and 10^3^ cells correspond to those with a higher number of reads in 10^6^ ESC DamID-seq (Fig. 4C), and they overlapped with ∼80% and ∼60% of mESC super enhancers (Whyte et al. 2013), respectively (Fig. 4D). The POU5F1-SOX2 motif and gene ontologies related to known POU5F1 functions (“stem cell maintenance,” “neural tube closure”) were enriched in 10^3^ ESC POU5F1 DamID-seq (Fig. 4E,F). Similarly SOX2 DamID-seq peaks identified in 10^4^ and 10^3^ NSCs highly overlapped with those in 10^6^ NSCs (Fig. 4G,H). The SOX2 motifs and NSC-related Gene Ontologies (“hindbrain development,” “neuronal stem cell maintenance”) were enriched in 10^3^ NSC SOX2 DamID-seq (Fig. 4I,J). In agreement with the fact that peaks from 10^4^ and 10^3^ cells overlap with stronger peaks in 10^6^-cell DamID-seq, a higher proportion of 10^4^- and 10^3^-cell DamID-seq peaks overlapped with ChIP-seq peaks (Supplemental Fig. S10A,B). Consistently, DamID-seq peaks from 10^4^ and 10^3^ are enriched in the overlapping peaks between 10^6^ DamID-seq and ChIP-seq peaks (Supplemental Fig. S10C,D). Thus, even when using lower cell numbers, DamID-seq specifically identifies the most robust binding sites, likely representing functionally relevant TF-genome engagement events. Overall, these data indicate the wide applicability of DamID-seq across different cell types and TFs, even when the starting cell number is limited. To our knowledge, these data represent the lowest number of cells used for the identification of TF binding sites in mammalian cells.

### POU5F1 DamID-seq in vivo

**Figure 5.**
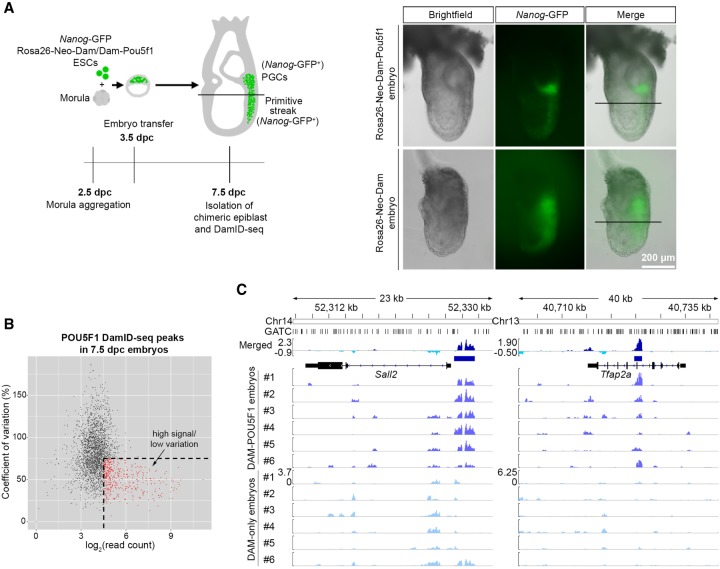
POU5F1 DamID-seq with 7.5-dpc epiblasts. (*A*) DamID-seq samples were prepared from each Dam/Dam-POU5F1–expressing 7.5-dpc chimeric embryo generated via morula aggregation, excluding the PGC-containing region. *Nanog*-GFP confirms contribution of Dam/Dam-POU5F1 ESCs, while the reporter expression is limited to posterior. (*B*) Read counts and coefficient of variation of the epiblast POU5F1 DamID-seq peaks. Peaks with high read counts (log_2_ > 4.5) and low standard deviation (<75%) indicated in red were used for further analyses. (*C*) The merged epiblast POU5F1 DamID-seq tracks (*top*) generated from six *Rosa26-Neo-Dam-Pou5f1* and six *Rosa26-Neo-Dam* embryos. Blue bars indicate selected confident peaks from *B*.

Next, we performed in vivo POU5F1 DamID-seq using ∼7.5 d post-coitum (dpc) embryos containing approximately 15,000 cells (Snow 1977; Tzouanacou et al. 2009), where endogenous POU5F1 is ubiquitously expressed (Rosner et al. 1990; Peng et al. 2016), by generating chimeric embryos with *Rosa26-Neo-Dam* and *Rosa26-Neo-Dam-Pou5f1* ESCs (Fig. 5A).

Each embryo showed a different level of ESC contribution as observed by the amount of *Nanog*-GFP reporter–positive ESC-derived cells in the posterior region of the embryos. The differentiation into multiple cell types within each embryo could further reduce the consistency of POU5F1 binding sites in the bulk samples. Accordingly, the in vivo DamID-seq data showed a higher variability between replicates compared with the POU5F1 ESC and SOX2 NSC data (Supplemental Fig. S11). Despite this variability, we identified 2768 peaks, which consistently had higher reads counts in six Dam-POU5F1–expressing embryos compared to six Dam-expressing embryos (FDR < 0.1), as seen for the 7356 peak regions identified in 1000 ESCs POU5F1 DamID-seq using four replicates each for Dam and Dam-POU5F1 (Supplemental Fig. S12).

**Figure 6.**
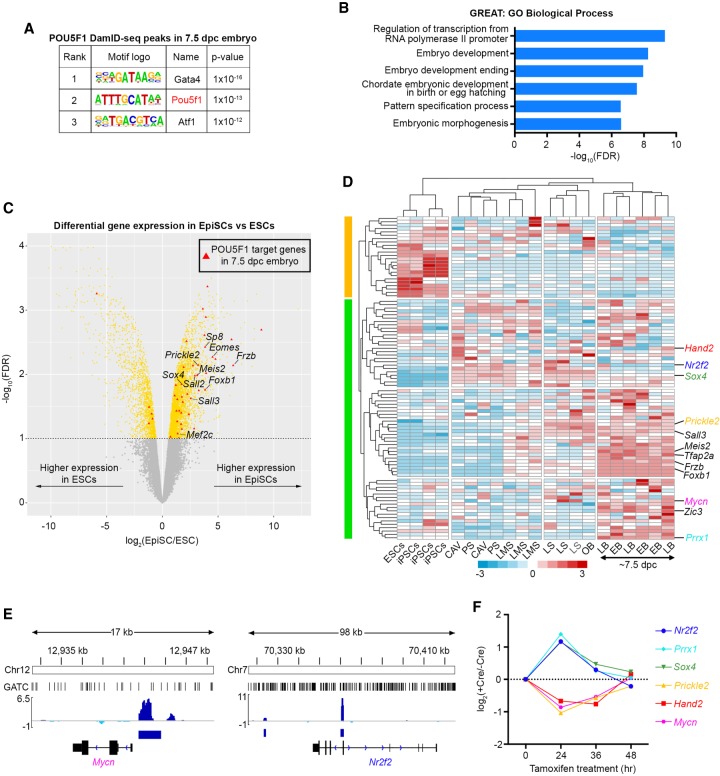
Analysis of POU5F1 target genes in the 7.5-dpc epiblasts. (*A*) Motif enrichment analysis (Heinz et al. 2010) of the epiblast POU5F1 DamID-seq peaks. (*B*) GO enrichment analysis of POU5F1 DamID-seq peaks from 7.5-dpc epiblasts using GREAT (McLean et al. 2010). (*C*) Differentially expressed genes between ESCs and EpiSCs (Tesar et al. 2007). Red triangles represent the POU5F1 binding peak–associated genes in 7.5-dpc epiblasts. (*D*) Expression levels of the 7.5-dpc epiblast POU5F1 binding peak–associated genes in ESCs and post-implantation epiblasts (Kojima et al. 2014). Genes whose expression significantly changed 24 h after *Pou5f1* deletion in the 7.5-dpc mouse embryo (DeVeale et al. 2013) are indicated in color. (CAV) Epiblast of cavity; (PS) prestreak; (LMS) late mid streak; (LS) late streak; (OB) no bud; (EB) early bud; (LB) late-bud. (*E*,*F*) POU5F1 binding peaks identified by DamID-seq (*E*) and expression changes of the development-related six genes upon *Pou5f1* deletion in 7.5-dpc embryos (*F*) (DeVeale et al. 2013).

We performed further analyses with the 343 high-confidence peaks with a high signal and lower variability among those 2768 peaks (Fig. 5B,C) and found the POU5F1 motif and GO terms related to early embryo development were highly enriched in those peaks and peak-associated genes, respectively (Fig. 6A,B).

Of the 94 ‘early embryo development’ peak-associated genes, many were highly expressed in EpiSCs, the in vitro counterpart of the 6- to 7.5-dpc epiblast (Tesar et al. 2007), rather than in ESCs (Fig. 6C). Consistently, in vivo transcriptome data (Kojima et al. 2014) demonstrated that most of these genes were up-regulated at post-implantation stages, in particular at the early bud (EB) and late bud (LB) stages (∼7.5 dpc) (green bar in Fig. 6D; Supplemental Table S2). It is noteworthy that the expression levels of the peak-associated genes, *Mycn*, *Hand2*, *Prickle2*, *Sox4*, *Prrx1*, and *Nr2f2*, changed approximately twofold 24 h after deletion of *Pou5f1* at ∼7.5 dpc using the Cre-loxP system (Fig. 6E,F; DeVeale et al. 2013). Thus, POU5F1 binding signatures identified by DamID-seq provide a valuable resource to elucidate how POU5F1 controls the expression of genes critical for embryo development (Osorno et al. 2012; DeVeale et al. 2013; Aires et al. 2016).

Overall, we optimized a powerful technique to identify TF–DNA interactions, DamID-seq, for mammalian cells and established it as a unique strategy to identify TF binding sites in limited numbers of cells including in vivo samples.

## Discussion

In this work, we have described an optimized DamID-seq for TF target identification in mouse cells by combining the use of the ubiquitously active endogenous *Gt(ROSA)26Sor* promoter with translation reinitiation. This approach has enabled us to detect TF targets in as few as 1000 cells and in developing mouse embryos at the gastrulation stage. The applicability of TF DamID-seq to much smaller cell numbers opens opportunities for new investigations in various biological contexts, even though the spatial resolution of DamID-seq is lower than ChIP-seq as the signal relies on the frequency of the GATC sequences (∼260 bp on average in the mouse genome). To date, ChIP-seq has been almost the only strategy used to uncover TF targets in a genome-wide manner in mammalian cells. Although several technical advances have been made (Furey 2012; Lara-Astiaso et al. 2014; Savic et al. 2015; Schmidl et al. 2015), TF ChIP-seq with more than 10,000 cells has not been reported.

Dam-POU5F1/Dam-SOX2 fusion proteins were expressed at extremely low levels using translation reinitiation, yet they labeled the binding sites of POU5F1 and SOX2, competing with these endogenously highly expressed TFs in ESCs and NSCs, respectively. The majority of stronger ChIP-seq peaks, which are supposed to have strong/more frequent binding of endogenous TFs, were identified by DamID-seq (Supplemental Fig. S9). Therefore, it is unlikely that the competition between lowly expressed exogenous Dam-POI and highly expressed endogenous POI compromises the identification of POI binding sites. In fact, DamID has been proven to be a powerful tool in *Drosophila* to uncover the binding sites of approximately 100 different chromatin proteins (Filion et al. 2010; van Bemmel et al. 2013), suggesting that this mouse translation-reinitiation DamID-seq can be applied to various TFs in different cell types in vitro and in vivo.

DamID has predominantly been used to investigate chromatin proteins (Filion et al. 2010; van Bemmel et al. 2013). For closed chromatin binding proteins, it is relatively straightforward to identify POI-specific binding because the background (Dam-only) signal is low, due to Dam's infrequent access to closed chromatin (Kladde and Simpson 1994). The first successful DamID experiments in human cells were performed for heterochromatin binding protein CBX1 and LMNB1 (Vogel et al. 2006; Guelen et al. 2008). More recently, DamID-seq has been used to investigate LMNB1 binding even in single human cells (Kind et al. 2015). In contrast, TF DamID has been more technically challenging since Dam-only frequently methylates GATC at open chromatin loci, resulting in high background methylation signal. In addition, TF binding sites are much narrower (8–20 bp) compared with lamina-associated domains (LADs; median size ∼0.5 Mb). Therefore, the number of DNA fragments obtained from one binding domain/site following DpnI digestion is much lower. This makes the DamID-seq peak calling step more difficult, which is based on the statistical comparison between Dam-only and Dam-TF–expressing cells. A limitation of DamID-seq is the requirement of the exogenous expression of Dam/Dam-POI. However, the PhiC31 RMCE system we have developed allows the generation of Dam-POI ESC lines via simple plasmid transfection. The resulting ESCs can be used to generate chimeric mice or mouse lines, making DamID-seq possible with any tissue of the mouse embryo since the *Gt(ROSA)26Sor* promoter is ubiquitously active.

Despite the constitutive expression of Dam/Dam-POU5F1, we could successfully detect gain and loss of TF binding signatures during cell differentiation since the methylation signal was most likely diluted through cell division. When investigating TF binding dynamics in post-mitotic cells or slowly dividing cells, a limitation of our DamID-seq system is the lack of inducibility of Dam-only/POI protein. A tightly regulated inducible tissue-specific Dam-only/POI protein expression system in adult mouse would further increase the applicability of DamID-seq in vivo (Southall et al. 2013; Pindyurin et al. 2016)*.* Another important development of TF DamID-seq would be its application in human cells, in which so far DamID has been used mainly for closed chromatin binding proteins by exploiting the leakiness of Ec promoter to express Dam/Dam-POI following viral delivery of the transgenes. In order to achieve a more controlled expression of Dam/Dam-POI and successful TF DamID-seq, human ES/iPS cell lines with the translation reinitiation system in a safe ubiquitously expressed locus (such as the AAVS1) could be established. Obtaining a large numbers of desired cell types in human ES/iPS cell differentiation is often difficult; thus, DamID-seq could have a large advantage over ChIP-seq in many experimental settings.

About 35% of POU5F1 peaks identified in three different ChIP-seq data sets were also identified by DamID-seq using a 10-fold lower number of mESCs. This overlap is substantial considering the intrinsic differences of the two techniques, the distinct data analysis methods, the variety of cell culture conditions, and the fact that the overlap between ChIP-seq data sets themselves is also limited. The features of DamID-seq–specific peaks were reminiscent of so-called “null” or “inert” chromatin previously described in *Drosophila melanogaster* (Filion et al. 2010; Kharchenko et al. 2011; Sexton et al. 2012), i.e., regions of the genome that are not enriched for any specific histone modification and characterized by a low transcriptional output. Dam-POU5F1/SOX2 binding signals in those regions might represent transient interactions of POU5F1/SOX2 with the genome, and their functional importance needs to be further investigated. Nevertheless, stronger POU5F1 DamID-seq peaks overlapped with the ChIP-seq peaks, as they probably represent highly robust and/or frequent binding events. Thus, selection of the strongest peaks among the total DamID peaks may enable one to select the peaks likely detectable in ChIP-seq. In summary, DamID-seq in mammalian cells represents a novel powerful tool to reveal targets of TFs in yet-unexplored biological contexts.

## Methods

### Vector construction

The coding sequence of Dam protein was taken from the pIND-V5-EcoDam (gift from Prof. Bas van Steensel, NKI). The *Neo-Dam*, *Neo-Dam-Pou5f1*, *Bsd-Dam*, *Bsd-Dam-Pou5f1*, *Hyg-Dam*, and *Hyg-Dam-Pou5f1 pENTR* vectors were generated by Gibson assembly (Gibson et al. 2009). Two stop codons (TAA TAA) were added at the end of each antibiotic-resistance gene, followed by a single C base and the ATG codon of *dam-only*/*dam-Pou5f1* (similar to the system described by Southall et al. 2013). A 16-amino-acid residue long linker (SGGGGSGGGGSGGGGS) was added between the *dam* and the *Pou5f1* coding region. *Neo-Dam* and *Neo-Dam-Pou5f1 pENTR* vectors were transferred into the *pROSA26-DEST* vector, after the *PGK-Neo* cassette was removed by in vitro Cre-loxP recombination, via Gateway LR II Clonase (Invitrogen) recombination. The dam* and dam*-Pou5f1 (dam* = D181A mutant) sequences were generated via site-directed mutagenesis and transferred into a *PB-CAG-pA-DEST* (gift from Prof. Andras Nagy, Mount Sinai Hospital) vector to generate CAG-Dam*-IRES-Puromycin-pA and CAG-Dam*-Pou5f1-IRES-Puromycin-pA. All the plasmids are available upon request.

### qDamID

gDNA was extracted from Dam-only/Dam-POU5F1 protein-expressing cells (about 1 × 10^6^ to 2 × 10^6^) using the DNeasy blood and tissue kit (Qiagen) according to the manufacturer's instructions. Extracted gDNA was treated overnight with and without DpnII enzyme, diluted to a final concentration of 2 ng/µL, of which 5 µL was used for each qPCR using the LightCycler480 instrument (Roche). The qPCR signal was converted to an absolute numerical value based on a standard curve*,* and the average of three triplicates from digested (+DpnII) and undigested (−DpnII) gDNA was used to calculate the percentage (%) of methylation at GATC_X_ site in Dam-only or Dam-POU5F1 expressing cells:

$${\%}_{{\mathrm{GATC}}_{X}}^{\mathrm{Dam}-\mathrm{only}\;\mathrm{or}\;\mathrm{Dam}-\mathrm{Pou}5f1}=\frac{\left[\text{Digested}\right]}{\left[\text{Undigested}\right]}\times 100.$$

This value was used to estimate the enrichment of Pou5f1 over Dam ${(\mathrm{DamID}}_{{\mathrm{GATC}}_{X}}^{\mathrm{ratio}})$, expressed as a subtraction:

$${\text{DamID}}_{{\mathrm{GATC}}_{X}}^{\mathrm{subtract}}=\frac{{\%}_{{\mathrm{GATC}}_{x}}^{\mathrm{Pou}5f1}}{\sum_{i}^{n}{\%}_{{\mathrm{GATC}}_{i}}^{\mathrm{Pou}5f1}}-\frac{{\%}_{{\mathrm{GATC}}_{x}}^{\mathrm{Dam}}}{\sum_{i}^{n}{\%}_{{\mathrm{GATC}}_{i}}^{\mathrm{Dam}}}.$$

The primers used for the qDamID are listed in the Supplemental Table S3.

### DamID-seq

All the DamID-seq experiments were performed in a minimum of three replicates for both Dam and Dam-POU5F1/SOX2. When using smaller number of cells (10^3^), we performed the experiments in four replicates. For the embryo data, we performed the experiments in six embryos each for Dam-only and Dam-POU5F1.

For DamID-seq experiments on cell lines, the desired number of cells were FAC-sorted using BD FACSAria II. When the number of cells was above 10,000, cells were first spun down and then resuspended in the genomic lysis buffer provided in the Quick-gDNA MicroPrep (ZymoResearch). When number of cells was lower than 10,000, cells were sorted directly into the genomic lysis buffer. The gDNA was extracted according to the manufacturer's instructions and eluted with 10 µL of the elution buffer. The gDNA was transferred into a 0.2-mL PCR tube containing 5 µL of DpnI mix (20 units DpnI, 1× CutSmart buffer [NEB]) and digested for 3 h at 37°C before heat inactivation of the enzyme for 20 min at 80°C. Double-strand DamID adapters (Vogel et al. 2007) were prepared by annealing AdRt (5′-CTAATACGACTCACTATAGGGCAGCGTGGTCGCGGCCGAGGA-3′, IDT) and AdRb (5′-TCCTCGGCCG-3′, IDT). Adapter ligation was performed by adding 5 µL of ligation mix (20 units T4 ligase, 1× T4 ligase buffer [NEB], and 0.2 µM DamID adapters) to the 15 µL of DpnI-digested DNA solution and keeping overnight at 16°C. After heat inactivation of ligase for 20 min at 65°C, 5 µL of DpnII mix (10 unit of DpnII and 1× DpnII buffer [NEB]) was added, and the samples were kept for 1 h at 37°C before heat inactivation for 20 min at 65°C. Then, 100 µL of PCR mix (KAPA HiFi HS ReadyMix [KAPA Biosystems, 1×], 10 µM AdR PCR primer, 5′-GGTCGCGGCCGAGGATC-3′[IDT], 1× SYBR green I nucleic acid gel stain [Life Technologies]) was added to the tube. From the final volume of 125 µL, 10 µL was used to perform qPCR in technical duplicate (in total 20 µL), and the number of PCR cycles to stop the PCR in the log-linear amplification phase was determined. The remaining 105 µL was then used to amplify the adapter-ligated fragments using the program displayed in Table 1.

**Table 1.**
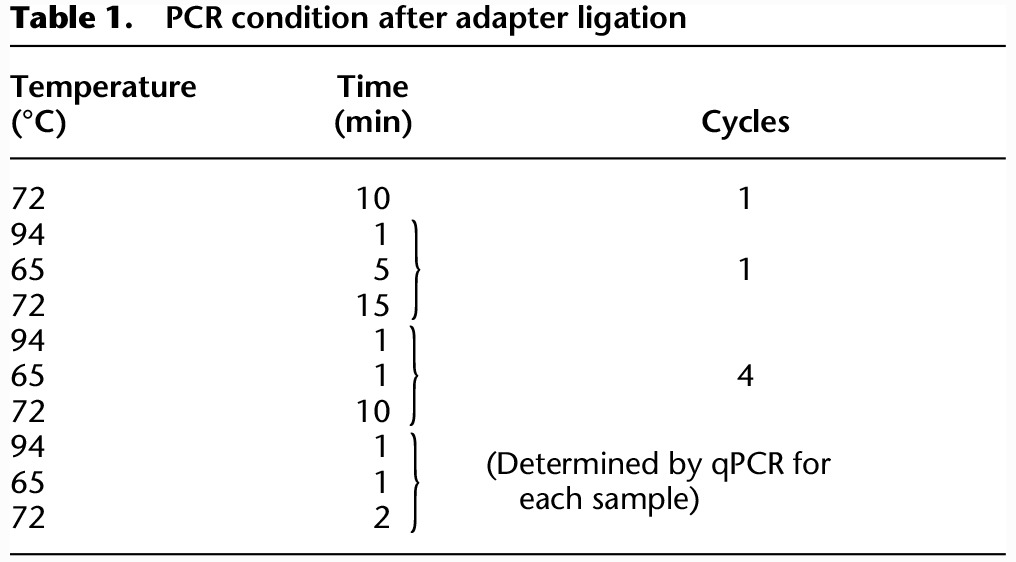
PCR condition after adapter ligation

Samples were purified using SPRI magnetic beads, resuspended in 30 µL H_2_O and quantified by Qubit 2.0 (Invitrogen). The sequencing libraries presented in this study have been prepared using the Nextera DNA sample preparation kit (Illumina), according to the manufacturer's instructions. Briefly, after Qubit quantification, 50 ng of the PCR amplified DNA was used in a tagmentation reaction (5 min) in which the Tn5 enzyme fragments the DNA and simultaneously inserts the preloaded Illumina adapters. After a DNA purification step using the DNA clean and concentrator kit (Zymo Research), the tagmented DNA was amplified by PCR for five cycles using the program displayed in Table 2.

**Table 2.**
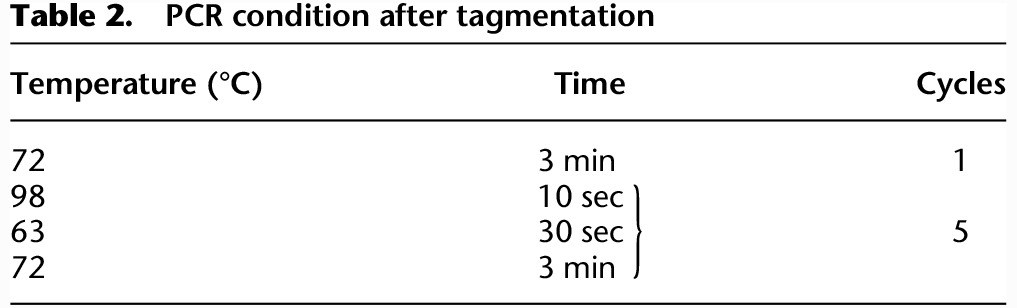
PCR condition after tagmentation

Primers used in the PCR amplification are barcoded, so a different pair of primers is used for each sample. After the PCR, DNA is purified using SPRI magnetic beads (0.8× DNA volume) and eluted in 30 µL of water. The purified DNA was used for Qubit 2.0 quantification and Tapestation analysis before pooling of the libraries and submission to the sequencing facility. The concentration of the library pool was usually ∼25–50 nM; 50-bp single-end sequencing was performed at Edinburgh Genomics on the Illumina HiSeq 2500 (chemistry v. 4) system or at BGI on the Illumina HiSeq 4000 system. A list of all the reagents and volumes required for each step of the newly optimized DamID-seq protocol (from gDNA extraction to next-generation library preparation) is summarized in Table 3.

**Table 3.**
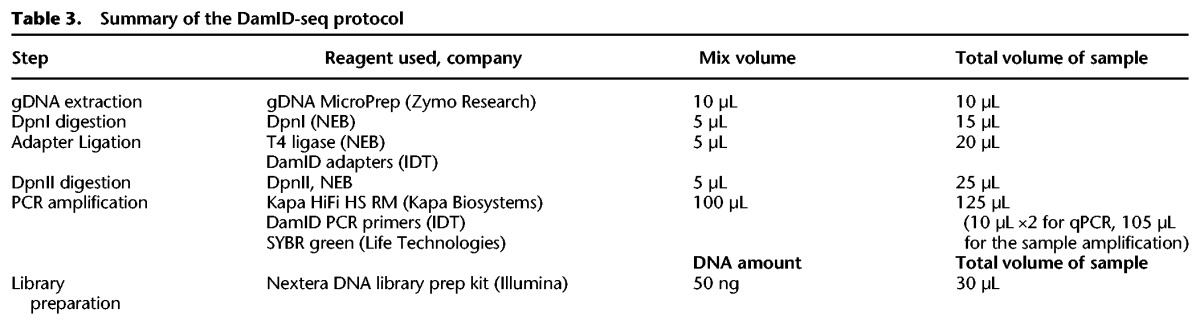
Summary of the DamID-seq protocol

### DamID-seq data analysis

#### Accession numbers

POU5F1 DamID-seq data in ESCs used for the comparison in Supplemental Figure S5 were downloaded from GEO with the GEO accession number GSE64008.

#### Sequencing data quality

Read quality was visualized using charts generated by FastQC (version 0.11.5) (http://www.bioinformatics.babraham.ac.uk/projects/fastqc/), and these charts were aggregated and visualized using MultiQC (version 0.9) (Ewels et al. 2016). The “per base sequence quality” chart showed reliable base calls across the entirety of the reads; the “sequence length distribution” chart confirmed that all our reads were of the same length; and the “sequence quality histogram” showed that the average quality per read was high (Phred scores: 30–40). Nextera sequencing adapters were removed using cutadapt and the relevant adapter sequence (version 1.13) (Martin 2011).

#### Sequencing data alignment

The trimmed reads were aligned to the UCSC mm10 assembly of the mouse genome (Church et al. 2009) using BWA MEM (version 0.7.15) (Li and Durbin 2010). For downstream analysis, we focused on just primary mapped reads; unmapped, secondary, and supplemental alignments were filtered using SAMtools (version 1.4) (Li et al. 2009). Reads mapped to uninformative (alternative, unplaced, and mitochondrial contigs) or ENCODE blacklist regions of the genome were also filtered using BEDTools (version 2.26.0) (Quinlan and Hall 2010). For all DamID-seq data, PCR duplicates were not removed. We reasoned that because reads were sequenced from distinct genomic features (GATC fragments), the probability of sequencing the same genomic region within a fragment would be high, and thus, the number of PCR duplicates would be overestimated. This is conceptually similar to RNA-seq analysis, where the probability of sequencing the exact same region from highly abundant transcripts is high.

#### Peak calling analysis

To identify binding sites from DamID-seq data, we measure how frequently each GATC fragment (windows along the genome enclosed by a GATC sequence) is bound by the Dam-only and Dam-POI proteins. Fragments that contain genuine binding sites will be bound by the Dam-POI protein more often than the Dam-only protein. Binding frequency is measured by sequencing fragments that have been methylated by the Dam protein, and differential binding is assessed by testing for significantly different read counts for each fragment between the Dam-only and Dam-POI samples.

Count matrices were created by counting the number of reads mapped fully within fragments, using the featureCounts command from Subread (version 1.5.0) (Liao et al. 2013). Low abundance fragments that correspond to background regions were subsequently removed to reduce the severity of the multiple testing correction, increase detection power among the remaining tests, and reduce computational load (Lun and Smyth 2016). An arbitrary threshold of 10 read counts or lower in equivalent average log-transformed counts per million (aveLogCPM) was used.

To address the high variability between DamID-seq replicates (particularly from low cell numbers), we applied smooth quantile normalization (Hicks et al. 2017). Unlike regular quantile normalization, this method does not assume that the observed variability in global properties are due only to technical reasons and are unrelated to the biology of interest (Hicks et al. 2017). The quantro package (version 1.8.0) (Hicks and Irizarry 2015) was used to test for global differences in the read count distributions between the Dam-only and Dam-POI samples and thus objectively measure the appropriateness of using smooth quantile normalization over regular quantile normalization. Normalized count matrices were created by generating logCPM values using the normalize function from the csaw package (version 1.8.1) (Lun and Smyth 2016) and then applying smooth quantile normalization to the logCPM values using the qsmooth package (version 0.0.1).

Differentially bound fragments were identified by testing for differential abundance between the Dam-only and Dam-POI samples. The normalized count matrices were given as input to the limma-trend function from the limma package (3.30.13). The trend and robust arguments were set to TRUE, fitting an intensity dependent trend to prior variances and applying the robust empirical Bayes procedure (Phipson et al. 2016). Differentially bound fragments were then combined into differentially bound peak regions using the mergeWindows and combineTests function from the csaw package. The tol and max.width arguments were set to 260 (median GATC fragment size for the mm10 assembly) and 10,000, respectively. Peaks were defined as regions with a false-discovery rate (FDR) smaller than 0.1 and a log fold-change (logFC) >0.5. All of the above peak calling analysis was carried out using the statistical programming language R (version 3.3.2) (R Core Team 2013) and packages from the Bioconductor project (version 3.4) (Huber et al. 2015).

#### Read coverage visualization

Read coverage across the genome was calculated with the genomecov command from BEDTools (version 2.26.0) (Quinlan and Hall 2010). Coverage for each sample was scaled using the normalization factors calculated in the peak calling analysis. The bedGraph files were converted into bigWig files with the UCSC bedGraphToBigWig (Kent et al. 2010) tool (version 4). Subtracted bigWig files were generated using the diff command from WiggleTools (version 1.2) (Zerbino et al. 2014).

### Downstream analysis of peak calls

The intersection between different sets of peaks was calculated using the tool mergePeaks of the HOMER suite (version 4.8.3) (Heinz et al. 2010). A proportional Venn diagram of three sets was generated using the software eulerAPE (version 3) (Micallef and Rodgers 2014), while proportional Venn diagrams of two sets were generated using the Venn diagram generator (http://jura.wi.mit.edu/bioc/tools/venn.php). Box-plots and scatter plots were generated using the package ggplot2 in the R statistical environment (version 3.3.2). The deepTools suite (Ramírez et al. 2016) was used to generate heatmaps using the computeMatrix and plotHeatmap commands.

## Data access

All DamID-seq data from this study have been submitted to the Gene Expression Omnibus (GEO; http://www.ncbi.nlm.nih.gov/geo/) under accession number GSE98092.

## Supplementary Material

Supplemental Material

## References

[GR227124TOSC1] Aires R, Jurberg AD, Leal F, Nóvoa A, Cohn MJ, Mallo M. 2016 Oct4 is a key regulator of vertebrate trunk length diversity. Dev Cell 38: 262–274. 27453501 10.1016/j.devcel.2016.06.021

[GR227124TOSC2] Ambrosetti DC, Basilico C, Dailey L. 1997 Synergistic activation of the fibroblast growth factor 4 enhancer by Sox2 and Oct-3 depends on protein–protein interactions facilitated by a specific spatial arrangement of factor binding sites. Mol Cell Biol 17: 6321–6329. 9343393 10.1128/mcb.17.11.6321 PMC232483

[GR227124TOSC3] Aughey GN, Southall TD. 2016 Dam it's good! DamID profiling of protein–DNA interactions. WIREs Dev Biol 5: 25–37. 10.1002/wdev.205 PMC4737221 26383089

[GR227124TOSC4] Baranello L, Kouzine F, Sanford S, Levens D. 2016 ChIP bias as a function of cross-linking time. Chromosome Res 24: 175–181. 26685864 10.1007/s10577-015-9509-1 PMC4860130

[GR227124TOSC5] Barski A, Cuddapah S, Cui K, Roh T-Y, Schones DE, Wang Z, Wei G, Chepelev I, Zhao K. 2007 High-resolution profiling of histone methylations in the human genome. Cell 129: 823–837. 17512414 10.1016/j.cell.2007.05.009

[GR227124TOSC6] Bouveret R, Waardenberg AJ, Schonrock N, Ramialison M, Doan T, de Jong D, Bondue A, Kaur G, Mohamed S, Fonoudi H, 2015 NKX2-5 mutations causative for congenital heart disease retain functionality and are directed to hundreds of targets. eLife 4: e06942. 10.7554/eLife.06942 PMC4548209 26146939

[GR227124TOSC7] Buecker C, Srinivasan R, Wu Z, Calo E, Acampora D, Faial T, Simeone A, Tan M, Swigut T, Wysocka J. 2014 Reorganization of enhancer patterns in transition from naive to primed pluripotency. Cell Stem Cell 14: 838–853. 24905168 10.1016/j.stem.2014.04.003 PMC4491504

[GR227124TOSC8] Church DM, Goodstadt L, Hillier LW, Zody MC, Goldstein S, She X, Bult CJ, Agarwala R, Cherry JL, DiCuccio M, 2009 Lineage-specific biology revealed by a finished genome assembly of the mouse. PLoS Biol 7: e1000112. 19468303 10.1371/journal.pbio.1000112 PMC2680341

[GR227124TOSC9] DeVeale B, Brokhman I, Mohseni P, Babak T, Yoon C, Lin A, Onishi K, Tomilin A, Pevny L, Zandstra PW, 2013 Oct4 is required ∼E7.5 for proliferation in the primitive streak. PLoS Genet 9: e1003957. 24244203 10.1371/journal.pgen.1003957 PMC3828132

[GR227124TOSC10] Ewels P, Magnusson M, Lundin S, Käller M. 2016 MultiQC: summarize analysis results for multiple tools and samples in a single report. Bioinformatics 32: 3047–3048. 27312411 10.1093/bioinformatics/btw354 PMC5039924

[GR227124TOSC11] Filion GJ, van Bemmel JG, Braunschweig U, Talhout W, Kind J, Ward LD, Brugman W, de Castro IJ, Kerkhoven RM, Bussemaker HJ, 2010 Systematic protein location mapping reveals five principal chromatin types in *Drosophila* cells. Cell 143: 212–224. 20888037 10.1016/j.cell.2010.09.009 PMC3119929

[GR227124TOSC12] Furey TS. 2012 ChIP–seq and beyond: new and improved methodologies to detect and characterize protein–DNA interactions. Nat Rev Genet 13: 840–852. 23090257 10.1038/nrg3306 PMC3591838

[GR227124TOSC13] Gibson DG, Young L, Chuang R-Y, Venter JC, Hutchison CA, Smith HO. 2009 Enzymatic assembly of DNA molecules up to several hundred kilobases. Nat Methods 6: 343–345. 19363495 10.1038/nmeth.1318

[GR227124TOSC14] Guelen L, Pagie L, Brasset E, Meuleman W, Faza MB, Talhout W, Eussen BH, de Klein A, Wessels L, de Laat W, 2008 Domain organization of human chromosomes revealed by mapping of nuclear lamina interactions. Nature 453: 948–951. 18463634 10.1038/nature06947

[GR227124TOSC15] Heinz S, Benner C, Spann N, Bertolino E, Lin YC, Laslo P, Cheng JX, Murre C, Singh H, Glass CK. 2010 Simple combinations of lineage-determining transcription factors prime *cis*-regulatory elements required for macrophage and B cell identities. Mol Cell 38: 576–589. 20513432 10.1016/j.molcel.2010.05.004 PMC2898526

[GR227124TOSC16] Hicks SC, Irizarry RA. 2015 quantro: a data-driven approach to guide the choice of an appropriate normalization method. Genome Biol 16: 117. 26040460 10.1186/s13059-015-0679-0 PMC4495646

[GR227124TOSC17] Hicks SC, Okrah K, Paulson JN, Quackenbush J, Irizarry RA, Bravo HC. 2017 Smooth quantile normalization. Biostatistics 19: 185–198. 10.1093/biostatistics/kxx028 PMC5862355 29036413

[GR227124TOSC18] Huber W, Carey VJ, Gentleman R, Anders S, Carlson M, Carvalho BS, Bravo HC, Davis S, Gatto L, Girke T, 2015 Orchestrating high-throughput genomic analysis with Bioconductor. Nat Methods 12: 115–121. 25633503 10.1038/nmeth.3252 PMC4509590

[GR227124TOSC19] Jacinto FV, Benner C, Hetzer MW. 2015 The nucleoporin Nup153 regulates embryonic stem cell pluripotency through gene silencing. Genes Dev 29: 1224–1238. 26080816 10.1101/gad.260919.115 PMC4495395

[GR227124TOSC20] Jain D, Baldi S, Zabel A, Straub T, Becker PB. 2015 Active promoters give rise to false positive “Phantom Peaks” in ChIP-seq experiments. Nucleic Acids Res 43: 6959–6968. 26117547 10.1093/nar/gkv637 PMC4538825

[GR227124TOSC21] Kent WJ, Zweig AS, Barber G, Hinrichs AS, Karolchik D. 2010 BigWig and BigBed: enabling browsing of large distributed datasets. Bioinformatics 26: 2204–2207. 20639541 10.1093/bioinformatics/btq351 PMC2922891

[GR227124TOSC22] Kharchenko PV, Alekseyenko AA, Schwartz YB, Minoda A, Riddle NC, Ernst J, Sabo PJ, Larschan E, Gorchakov AA, Gu T, 2011 Comprehensive analysis of the chromatin landscape in *Drosophila melanogaster*. Nature 471: 480–485. 21179089 10.1038/nature09725 PMC3109908

[GR227124TOSC23] Kind J, Pagie L, de Vries SS, Nahidiazar L, Dey SS, Bienko M, Zhan Y, Lajoie B, de Graaf CA, Amendola M, 2015 Genome-wide maps of nuclear lamina interactions in single human cells. Cell 163: 134–147. 26365489 10.1016/j.cell.2015.08.040 PMC4583798

[GR227124TOSC24] King HW, Klose RJ. 2017 The pioneer factor OCT4 requires the chromatin remodeller BRG1 to support gene regulatory element function in mouse embryonic stem cells. eLife 6: e22631. 28287392 10.7554/eLife.22631 PMC5400504

[GR227124TOSC25] Kladde MP, Simpson RT. 1994 Positioned nucleosomes inhibit Dam methylation in vivo. Proc Natl Acad Sci 91: 1361–1365. 8108416 10.1073/pnas.91.4.1361 PMC43158

[GR227124TOSC26] Kojima Y, Kaufman-Francis K, Studdert JB, Steiner KA, Power MD, Loebel DAF, Jones V, Hor A, de Alencastro G, Logan GJ, 2014 The transcriptional and functional properties of mouse epiblast stem cells resemble the anterior primitive streak. Cell Stem Cell 14: 107–120. 24139757 10.1016/j.stem.2013.09.014

[GR227124TOSC27] Kozak M. 2001 Constraints on reinitiation of translation in mammals. Nucleic Acids Res 29: 5226–5232. 11812856 10.1093/nar/29.24.5226 PMC97554

[GR227124TOSC28] Lara-Astiaso D, Weiner A, Lorenzo-Vivas E, Zaretsky I, Jaitin DA, David E, Keren-Shaul H, Mildner A, Winter D, Jung S, 2014 Immunogenetics. Chromatin state dynamics during blood formation. Science 345: 943–949. 25103404 10.1126/science.1256271 PMC4412442

[GR227124TOSC29] Li H, Durbin R. 2010 Fast and accurate long-read alignment with Burrows–Wheeler transform. Bioinformatics 26: 589–595. 20080505 10.1093/bioinformatics/btp698 PMC2828108

[GR227124TOSC30] Li H, Handsaker B, Wysoker A, Fennell T, Ruan J, Homer N, Marth G, Abecasis G, Durbin R, 1000 Genome Project Data Processing Subgroup. 2009 The Sequence Alignment/Map format and SAMtools. Bioinformatics 25: 2078–2079. 19505943 10.1093/bioinformatics/btp352 PMC2723002

[GR227124TOSC31] Liao Y, Smyth GK, Shi W. 2013 The Subread aligner: fast, accurate and scalable read mapping by seed-and-vote. Nucleic Acids Res 41: e108. 23558742 10.1093/nar/gkt214 PMC3664803

[GR227124TOSC32] Lun ATL, Smyth GK. 2016 csaw: a Bioconductor package for differential binding analysis of ChIP-seq data using sliding windows. Nucleic Acids Res 44: e45. 26578583 10.1093/nar/gkv1191 PMC4797262

[GR227124TOSC33] Marson A, Levine SS, Cole MF, Frampton GM, Brambrink T, Johnstone S, Guenther MG, Johnston WK, Wernig M, Newman J, 2008 Connecting microRNA genes to the core transcriptional regulatory circuitry of embryonic stem cells. Cell 134: 521–533. 18692474 10.1016/j.cell.2008.07.020 PMC2586071

[GR227124TOSC34] Martin M. 2011 Cutadapt removes adapter sequences from high-throughput sequencing reads. EMBnetjournal 17: 10.

[GR227124TOSC35] Mateo JL, van den Berg DLC, Haeussler M, Drechsel D, Gaber ZB, Castro DS, Robson P, Crawford GE, Flicek P, Ettwiller L, 2015 Characterization of the neural stem cell gene regulatory network identifies OLIG2 as a multifunctional regulator of self-renewal. Genome Res 25: 41–56. 25294244 10.1101/gr.173435.114 PMC4317172

[GR227124TOSC36] McLean CY, Bristor D, Hiller M, Clarke SL, Schaar BT, Lowe CB, Wenger AM, Bejerano G. 2010 GREAT improves functional interpretation of *cis*-regulatory regions. Nat Biotechnol 28: 495–501. 20436461 10.1038/nbt.1630 PMC4840234

[GR227124TOSC37] Micallef L, Rodgers P. 2014 eulerAPE: drawing area-proportional 3-Venn diagrams using ellipses. PLoS One 9: e101717. 25032825 10.1371/journal.pone.0101717 PMC4102485

[GR227124TOSC38] Mistri TK, Devasia AG, Chu LT, Ng WP, Halbritter F, Colby D, Martynoga B, Tomlinson SR, Chambers I, Robson P, 2015 Selective influence of Sox2 on POU transcription factor binding in embryonic and neural stem cells. EMBO Rep 16: 1177–1191. 26265007 10.15252/embr.201540467 PMC4576985

[GR227124TOSC39] Moorman C, Sun LV, Wang J, de Wit E, Talhout W, Ward LD, Greil F, Lu X-J, White KP, Bussemaker HJ, 2006 Hotspots of transcription factor colocalization in the genome of *Drosophila melanogaster*. Proc Natl Acad Sci 103: 12027–12032. 16880385 10.1073/pnas.0605003103 PMC1567692

[GR227124TOSC40] Niwa H, Miyazaki J-I, Smith A. 2000 Quantitative expression of Oct-3/4 defines differentiation, dedifferentiation or self-renewal of ES cells. Nat Genet 24: 372–376. 10742100 10.1038/74199

[GR227124TOSC41] Osorno R, Tsakiridis A, Wong F, Cambray N, Economou C, Wilkie R, Blin G, Scotting PJ, Chambers I, Wilson V. 2012 The developmental dismantling of pluripotency is reversed by ectopic Oct4 expression. Development 139: 2288–2298. 22669820 10.1242/dev.078071 PMC3367440

[GR227124TOSC42] Park D, Lee Y, Bhupindersingh G, Iyer VR. 2013 Widespread misinterpretable ChIP-seq bias in yeast. PLoS One 8: e83506. 24349523 10.1371/journal.pone.0083506 PMC3857294

[GR227124TOSC43] Peng G, Suo S, Chen J, Chen W, Liu C, Yu F, Wang R, Chen S, Sun N, Cui G, 2016 Spatial transcriptome for the molecular annotation of lineage fates and cell identity in mid-gastrula mouse embryo. Dev Cell 36: 681–697. 27003939 10.1016/j.devcel.2016.02.020

[GR227124TOSC44] Phipson B, Lee S, Majewski IJ, Alexander WS, Smyth GK. 2016 Robust hyperparameter estimation protects against hypervariable genes and improves power to detect differential expression. Ann Appl Stat 10: 946–963. 28367255 10.1214/16-AOAS920 PMC5373812

[GR227124TOSC45] Picelli S, Björklund ÅK, Reinius B, Sagasser S, Winberg G, Sandberg R. 2014 Tn5 transposase and tagmentation procedures for massively scaled sequencing projects. Genome Res 24: 2033–2040. 25079858 10.1101/gr.177881.114 PMC4248319

[GR227124TOSC46] Pindyurin AV, Pagie L, Kozhevnikova EN, van Arensbergen J, van Steensel B. 2016 Inducible DamID systems for genomic mapping of chromatin proteins in *Drosophila*. Nucleic Acids Res 44: 5646–5657. 27001518 10.1093/nar/gkw176 PMC4937306

[GR227124TOSC47] Quinlan AR, Hall IM. 2010 BEDTools: a flexible suite of utilities for comparing genomic features. Bioinformatics 26: 841–842. 20110278 10.1093/bioinformatics/btq033 PMC2832824

[GR227124TOSC48] R Core Team. 2013 R: a language and environment for statistical computing. R Foundation for Statistical Computing, Vienna, Austria http://www.R-project.org/.

[GR227124TOSC49] Ramírez F, Ryan DP, Grüning B, Bhardwaj V, Kilpert F, Richter AS, Heyne S, Dündar F, Manke T. 2016 deepTools2: a next generation web server for deep-sequencing data analysis. Nucleic Acids Res 44: W160–W165. 27079975 10.1093/nar/gkw257 PMC4987876

[GR227124TOSC50] Rosner MH, Vigano MA, Ozato K, Timmons PM, Poirier F, Rigby PW, Staudt LM. 1990 A POU-domain transcription factor in early stem cells and germ cells of the mammalian embryo. Nature 345: 686–692. 1972777 10.1038/345686a0

[GR227124TOSC51] Savic D, Partridge EC, Newberry KM, Smith SB, Meadows SK, Roberts BS, Mackiewicz M, Mendenhall EM, Myers RM. 2015 CETCh-seq: CRISPR epitope tagging ChIP-seq of DNA-binding proteins. Genome Res 25: 1581–1589. 26355004 10.1101/gr.193540.115 PMC4579343

[GR227124TOSC52] Schmidl C, Rendeiro AF, Sheffield NC, Bock C. 2015 ChIPmentation: fast, robust, low-input ChIP-seq for histones and transcription factors. Nat Methods 12: 963–965. 26280331 10.1038/nmeth.3542 PMC4589892

[GR227124TOSC53] Sexton T, Yaffe E, Kenigsberg E, Bantignies F, Leblanc B, Hoichman M, Parrinello H, Tanay A, Cavalli G. 2012 Three-dimensional folding and functional organization principles of the *Drosophila* genome. Cell 148: 458–472. 22265598 10.1016/j.cell.2012.01.010

[GR227124TOSC54] Simandi Z, Horvath A, Wright LC, Cuaranta-Monroy I, De Luca I, Karolyi K, Sauer S, Deleuze J-F, Gudas LJ, Cowley SM, 2016 OCT4 acts as an integrator of pluripotency and signal-induced differentiation. Mol Cell 63: 647–661. 27499297 10.1016/j.molcel.2016.06.039

[GR227124TOSC55] Snow M. 1977 Gastrulation in the mouse: growth and regionalization of the epiblast. J Embryol Exp Morphol 42: 293–303.

[GR227124TOSC56] Soufi A, Garcia MF, Jaroszewicz A, Osman N, Pellegrini M, Zaret KS. 2015 Pioneer transcription factors target partial DNA motifs on nucleosomes to initiate reprogramming. Cell 161: 555–568. 25892221 10.1016/j.cell.2015.03.017 PMC4409934

[GR227124TOSC57] Southall TD, Gold KS, Egger B, Davidson CM, Caygill EE, Marshall OJ, Brand AH. 2013 Cell-type-specific profiling of gene expression and chromatin binding without cell isolation: assaying RNA Pol II occupancy in neural stem cells. Dev Cell 26: 101–112. 23792147 10.1016/j.devcel.2013.05.020 PMC3714590

[GR227124TOSC58] Steube A, Schenk T, Tretyakov A, Saluz HP. 2017 High-intensity UV laser ChIP-seq for the study of protein–DNA interactions in living cells. Nat Commun 8: 1303. 29101361 10.1038/s41467-017-01251-7 PMC5670203

[GR227124TOSC59] Tesar PJ, Chenoweth JG, Brook FA, Davies TJ, Evans EP, Mack DL, Gardner RL, McKay RDG. 2007 New cell lines from mouse epiblast share defining features with human embryonic stem cells. Nature 448: 196–199. 17597760 10.1038/nature05972

[GR227124TOSC60] Teytelman L, Thurtle DM, Rine J, van Oudenaarden A. 2013 Highly expressed loci are vulnerable to misleading ChIP localization of multiple unrelated proteins. Proc Natl Acad Sci 110: 18602–18607. 24173036 10.1073/pnas.1316064110 PMC3831989

[GR227124TOSC61] Tzouanacou E, Wegener A, Wymeersch FJ, Wilson V, Nicolas J-F. 2009 Redefining the progression of lineage segregations during mammalian embryogenesis by clonal analysis. Dev Cell 17: 365–376. 19758561 10.1016/j.devcel.2009.08.002

[GR227124TOSC62] Urig S, Gowher H, Hermann A, Beck C, Fatemi M, Humeny A, Jeltsch A. 2002 The *Escherichia coli* dam DNA methyltransferase modifies DNA in a highly processive reaction. J Mol Biol 319: 1085–1096. 12079349 10.1016/S0022-2836(02)00371-6

[GR227124TOSC63] van Bemmel JG, Filion GJ, Rosado A, Talhout W, de Haas M, van Welsem T, van Leeuwen F, van Steensel B. 2013 A network model of the molecular organization of chromatin in *Drosophila*. Mol Cell 49: 759–771. 23438860 10.1016/j.molcel.2013.01.040

[GR227124TOSC64] van Steensel B, Delrow J, Henikoff S. 2001 Chromatin profiling using targeted DNA adenine methyltransferase. Nat Genet 27: 304–308. 11242113 10.1038/85871

[GR227124TOSC65] Vogel MJ, Guelen L, de Wit E, Peric-Hupkes D, Lodén M, Talhout W, Feenstra M, Abbas B, Classen A-K, van Steensel B. 2006 Human heterochromatin proteins form large domains containing KRAB-ZNF genes. Genome Res 16: 1493–1504. 17038565 10.1101/gr.5391806 PMC1665633

[GR227124TOSC66] Vogel MJ, Peric-Hupkes D, van Steensel B. 2007 Detection of *in vivo* protein–DNA interactions using DamID in mammalian cells. Nat Protoc 2: 1467–1478. 17545983 10.1038/nprot.2007.148 PMC7032955

[GR227124TOSC67] Whyte WA, Orlando DA, Hnisz D, Abraham BJ, Lin CY, Kagey MH, Rahl PB, Lee TI, Young RA. 2013 Master transcription factors and mediator establish super-enhancers at key cell identity genes. Cell 153: 307–319. 23582322 10.1016/j.cell.2013.03.035 PMC3653129

[GR227124TOSC68] Wines DR, Talbert PB, Clark DV, Henikoff S. 1996 Introduction of a DNA methyltransferase into Drosophila to probe chromatin structure in vivo. Chromosoma 104: 332–340. 8575244 10.1007/BF00337221

[GR227124TOSC69] Wreczycka K, Franke V, Uyar B, Wurmus R, Akalin A. 2017 HOT or not: examining the basis of high-occupancy target regions. bioRxiv 10.1101/107680. PMC6582337 31114922

[GR227124TOSC70] Zerbino DR, Johnson N, Juettemann T, Wilder SP, Flicek P. 2014 WiggleTools: parallel processing of large collections of genome-wide datasets for visualization and statistical analysis. Bioinformatics 30: 1008–1009. 24363377 10.1093/bioinformatics/btt737 PMC3967112

