# Supplementary material for: Mapping transcription factor occupancy using minimal numbers of cells in vitro and in vivo
Source: Genome Res. 2018 Apr;28(4):592–605. doi: 10.1101/gr.227124.117 (PMC5880248; doi:10.1101/gr.227124.117)
Supplement: Supplemental Material [file supp_28_4_592__index.html]

Mapping transcription factor occupancy using minimal numbers of cells in vitro and in vivo — Mapping transcription factor occupancy using minimal numbers of cells in vitro and in vivo — Supplemental Material 

# Mapping transcription factor occupancy using minimal numbers of cells in vitro and in vivo

## Supplemental Material

- Supplemental\_Table\_S3.docx
- Supplemental\_Fig\_S1.pdf
- Supplemental\_Fig\_S2.pdf
- Supplemental\_Fig\_S3.pdf
- Supplemental\_Fig\_S4.pdf
- Supplemental\_Fig\_S5.pdf
- Supplemental\_Fig\_S6.pdf
- Supplemental\_Fig\_S7.pdf
- Supplemental\_Fig\_S8.pdf
- Supplemental\_Fig\_S9.pdf
- Supplemental\_Fig\_S10.pdf
- Supplemental\_Fig\_S11.pdf
- Supplemental\_Fig\_S12.pdf
- Supplemental\_Material\_S1.tar.gz
- Supplemental\_Methods.docx
- Supplemental\_Table\_S1.docx
- Supplemental\_Table\_S2.xlsx
