# Supplementary material for: Mapping transcription factor occupancy using minimal numbers of cells in vitro and in vivo
Source: Genome Res. 2018 Apr;28(4):592–605. doi: 10.1101/gr.227124.117 (PMC5880248; doi:10.1101/gr.227124.117)
Supplement: Supplemental Material [file supp_gr.227124.117_Supplemental_Table_S3.docx]

| **Southern Blot probes** | | | | |
| --- | --- | --- | --- | --- |
| **Probe** | **Forward (5’->3’)** | | | **Reverse (5’->3’)** |
| *Rosa26* | CAAGTGCTCCATGCTGGAAGGATTG | | | TGATTGGGGAGGATCCAGATGGAG |
| *Neo* | GGATTGCACGCAGGTTCTCCG | | | CGCCGCCAAGCTCTTCAGCAA |
| *Bsd* | ATGCCTTTGTCTCAAGAAGAATCCACC | | | TTAACCCTCCCACACATAACCAGAGG |
| **RMCE - Genomic PCR** | | | | |
| **Primer** | **Forward (5’->3’)** | **Reverse (5’->3’)** | | |
| *Hyg 5’* | AGGACAAACTCTTCGCGGTCTTTC | TGAGTTCAGGCTTTCCGGATCTATCCAT | | |
| *Dam 3’* | TGCTGGCTTTGTACAAACCAGGAG | TGACACCTACTCAGACAATGCGA | | |
| *Pou5f1 3’* | GGCTCTCCCATGCATTCAAACTGA | Same as *Dam 3’* reverse primer | | |
| **Gene expression analysis** | | | | |
| **Gene** | **Forward (5’->3’)** | **Reverse (5’->3’)** | | |
| *Pou5f1* | GTTGGAGAAGGTGGAACCAA | CTCCTTCTGCAGGGCTTTC | | |
| *Nanog* | CCTCCAGCAGATGCAAGAA | GCTTGCACTTCATCCTTTGG | | |
| *Esrrb* | CGATTCATGAAATGCCTCAA | CCTCCTCGAACTCGGTCA | | |
| *Rex1* | ACGAGTGGCAGTTTCTTCTTGGGA | TATGACTCACTTCCAGGGGGCACT | | |
| *Klf4* | AAGAACAGCCACCCACAC TT | GGTAAGGTTTCTCGCCTGTG | | |
| *Sox2* | GGCGGCAACCAGAAGAACAG | GCTTGGCCTCGTCGATGAAC | | |
| *Otx2* | CCACTTCGGGTATGGACTTG | GTCCTCTCCCTTCGCTGTTT | | |
| *Tbp* | GGGGAGCTGTGATGTGAAGT | CCAGGAAATAATTCTGGCTCA | | |
| *Gapdh* | CCCACTAACATCAAATGGGG | CCTTCCACAATGCCAAAGTT | | |
| *Tbx3* | GCATCCTCTCCTGCTGTCTC | GCCGTAGTGGTGGAAATCTT | | |
| *Dam* | GTGTTTCTCAACACCGACTTTTCT | ACTCATCAGTACGCATCTTCACAA | | |
| **qDamID analysis** | | | | |
| **Target** | **Forward (5’->3’)** | | **Reverse (5’->3’)** | |
| Oct4_1 | GCAATCGTGTCAATGGAAGA | | CACAGCATTAGAACCACCCA | |
| Oct4_2 | ATGCTGGTGGACCATCTCTG | | CAGGTGGTTTGTGGCAAGTT | |
| Oct4_3 | CCCAGGAGGCAAGAAACTG | | AGGGCACATCTGTTTCAAGCT | |
| Oct4_4 | CTTGCAGACAGGCACTCTGA | | TCCTCCTAATCCCGTCTCCT | |
| Oct4_5 | GACCAGGCTCAGAGGTATTGG | | GAAAGCAACTCAGAGGGAACCT | |
| Oct4_6 | ACCCTTGGGTAGGGGAGTTT | | AAGAACAAGGTGCGAGTGGA | |
| Oct4_7 | GGCATGGACATTTGGCTACT | | ATTACCCCAGCCCTTGAGAC | |
| Oct4_8 | GCGTTCTCTTTGGTGGGTCT | | TGCTTCTCCACAGGTAAGGG | |
| Oct4_9 | CCATCTCTGTGCCCATCCTA | | TACAGGGACACCTTTCCCAG | |
| Has2_1 | AATGGGGTAAGAATGGGGTTCT | | GTGATACAAGGGCAAGGAGGTT | |
| Has2_2 | TGCAGCATGAGGAAGTGTGTTA | | TAGGGTTGGGAAAACCTCAAGA | |
| Has2_3 | AAAATCCTTGCCACAGAAGCTC | | GGGGGTGTGCTTGTAGATTCTC | |
| Has2_4 | CAAATGGTCTAAACTGGAGAGTGA | | CTATTTCAAGGAAGGGGCTGGT | |
| Has2_5 | CGCTGCCCAAATCCAATATCTA | | TCTGAAAGCCAAAAGCACATTC | |
| Tcl1_1 | TATGCAGGACAACAACGTGGAC | | CATAGGGCAGTGTTTCCTGACA | |
| Tcl1_2 | GAAGCCGTTGGTGGTCTTTGG | | CCCAAGTGCTGTGATTAAAGGCA | |
| Tcl1_3 | TAGAGGTGTGAGTGCCCATGTG | | CCCAGACCCCAGCTTAGAGGT | |

Supplemental Table S3 – Primer list
