# Supplementary material for: Mapping transcription factor occupancy using minimal numbers of cells in vitro and in vivo
Source: Genome Res. 2018 Apr;28(4):592–605. doi: 10.1101/gr.227124.117 (PMC5880248; doi:10.1101/gr.227124.117)
Supplement: Supplemental Material [file supp_gr.227124.117_Supplemental_Fig_S1.pdf]

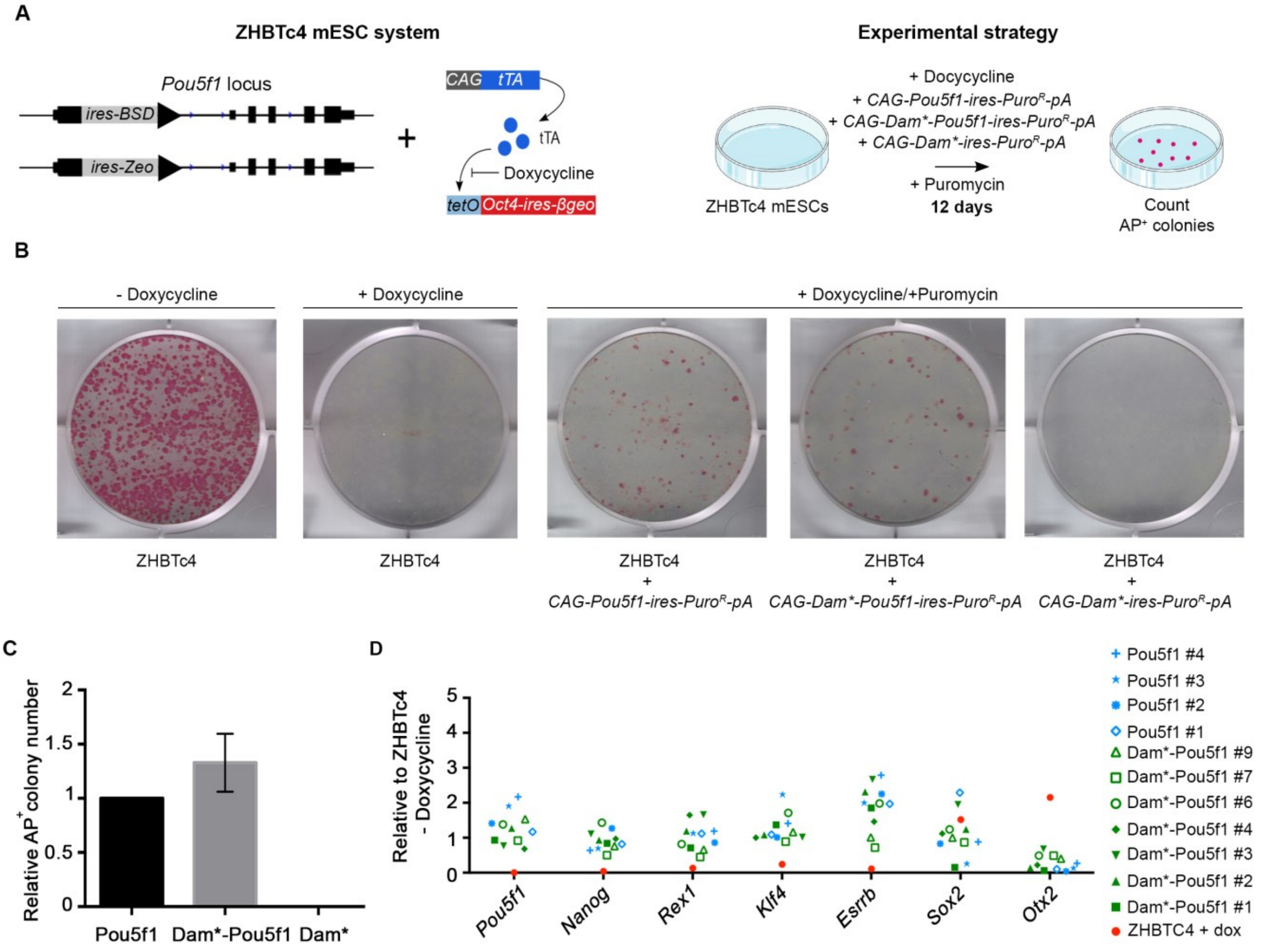

Supplemental Figure S1: DAM-POU5F1 is functionally equivalent to POU5F1. (*A*) In ZHBTc4 ESCs, two endogenous *Pou5f1* alleles were knocked out, and tetO promoter-driven POU5F1 sustains an undifferentiated state in the absence of doxycycline (Niwa et al. 2000) (left). The ability to maintain the undifferentiated state of POU5F1, DAM*-POU5F1 and DAM* expression vector was assessed upon administration of doxycycline (right). *Dam** (Dam D181A mutant), which lacks methylation activity without altering its DNA binding capacity (Liebert et al. 2004), was used in these experiments as over-expression of wild-type DAM is toxic. (*B*) Alkaline phosphatase (AP) staining identified undifferentiated ZHBTc4 ESC colonies maintained by wild type POU5F1 and DAM*-POU5F1. (*C*) Quantification of AP^+ colonies in ZHBTc4 ESCs transfected with POU5F1 wild type, DAM*-POU5F1 or DAM*-only. Data represent mean ± s.e.m. (n=2). (*D*) Gene expression of ZHBTc4 ESC clones (passage 5) maintained with POU5F1 wild type (blue), DAM*-POU5F1 (green), and differentiated cells without a rescue plasmid (red).
