# Supplementary material for: Mapping transcription factor occupancy using minimal numbers of cells in vitro and in vivo
Source: Genome Res. 2018 Apr;28(4):592–605. doi: 10.1101/gr.227124.117 (PMC5880248; doi:10.1101/gr.227124.117)
Supplement: Supplemental Material [file supp_gr.227124.117_Supplemental_Fig_S2.pdf]

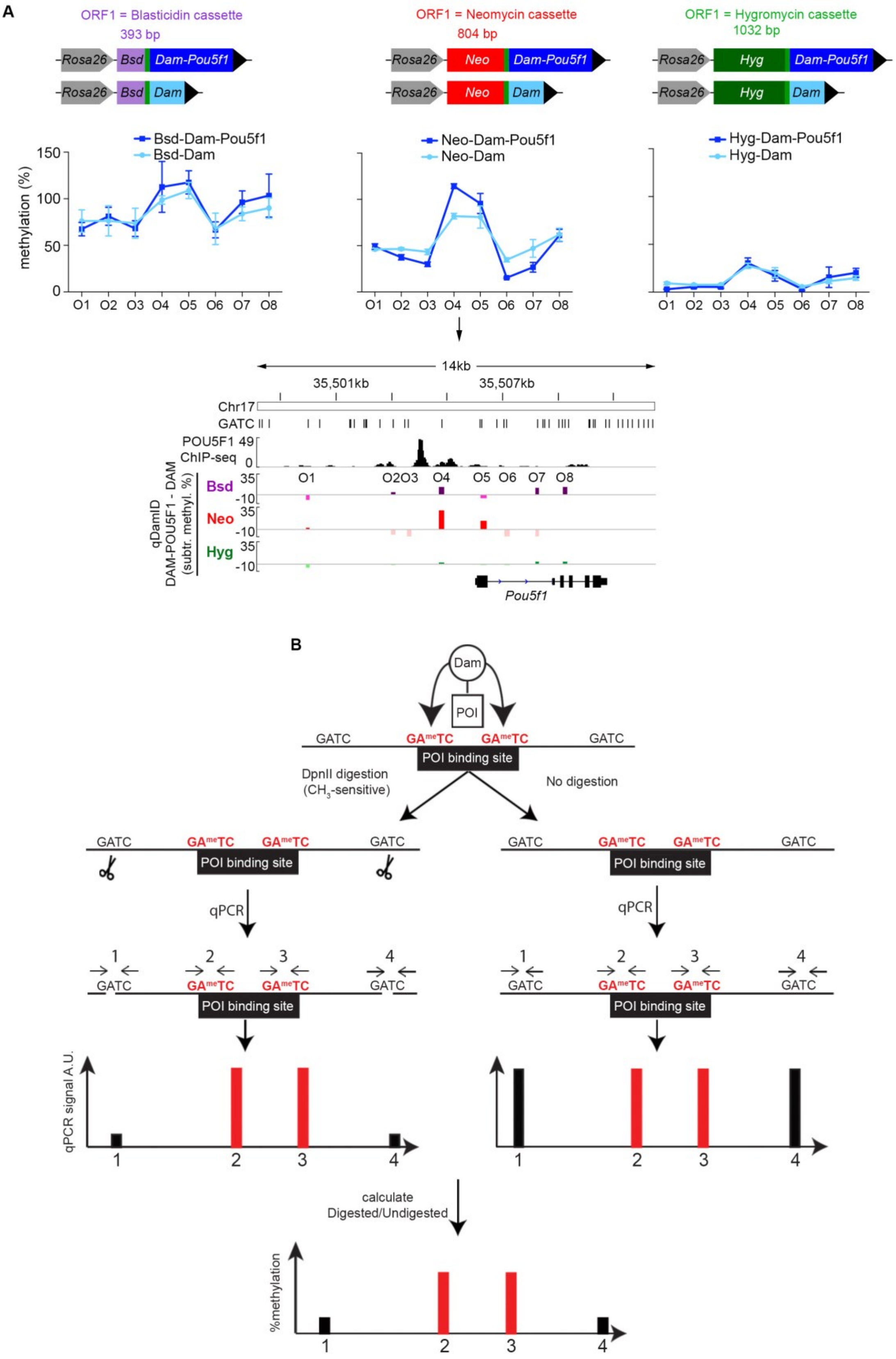

Supplemental Figure S2: Identification of the optimal 1st ORF for translation reinitiation-mediated DamID. (*A*) Methylation levels in the *Pou5f1* locus measured by qDamID (see *B*) in cell lines with different 1st ORFs before the *Dam*/*Dam-Pou5f1* coding sequences. Data represent mean ± s.e.m. (n=4). O1-O8 represents the position of the GATC sites analysed by qDamID. The methylation levels (%) in DAM-POU5F1, DAM expressing cell lines were used to calculate the subtraction (DAM-POU5F1)-(DAM). *Bsd* = blasticidin resistance gene; *Neo* = neomycin resistance gene; *Hyg* = hygromycin resistance gene. (*B*) Schematic representation of the qDamID workflow. POI = protein of interest; GA^meTC = adenine-methylated GATC sequence; A.U. = arbitrary unit.
