# Supplementary material for: Mapping transcription factor occupancy using minimal numbers of cells in vitro and in vivo
Source: Genome Res. 2018 Apr;28(4):592–605. doi: 10.1101/gr.227124.117 (PMC5880248; doi:10.1101/gr.227124.117)
Supplement: Supplemental Material [file supp_gr.227124.117_Supplemental_Fig_S3.pdf]

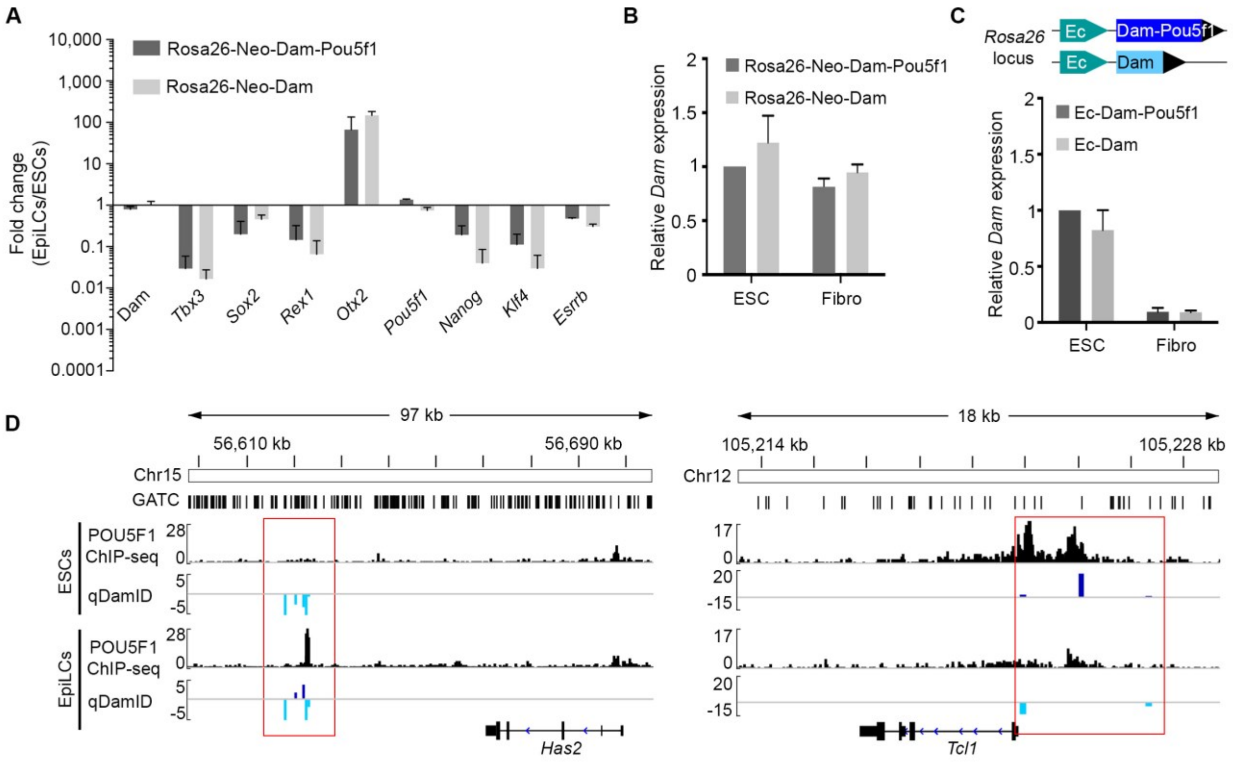

Supplemental Figure S3: Gain and loss of qDamID signal upon ESCs differentiation. (*A*) Similar expression changes of pluripotency-related genes after conversion of Rosa26-Neo-Dam-Pou5f1 and Rosa26-Neo-Dam ESCs to EpiLCs. Data plotted as mean ± s.e.m. (n=3). (*B*) Expression level of *Dam* in Rosa26-Neo-Dam-Pou5f1 and Rosa26-Neo-Dam ESCs and *in vitro* differentiated fibroblasts. Data represent mean ± s.e.m. (n=2). (*C*) Expression level of *Dam* mRNA in Rosa26-Ec-Dam-Pou5f1 and Rosa26-Ec-Dam ESCs and *in vitro* differentiated fibroblasts. Data represent mean ± s.e.m. (n=2). Ec = Ecdysone-inducible promoter. (*D*) qDamID analysis in the *Has2* and *Tcl1* loci with Rosa26-Neo-Dam-Pou5f1 and Rosa-26-Neo-Dam ESCs and EpiLCs in comparison with POU5F1 ChIP-seq data (Buecker et al. 2014). Y-axis; subtracted methylation level (%).
