# Supplementary material for: Mapping transcription factor occupancy using minimal numbers of cells in vitro and in vivo
Source: Genome Res. 2018 Apr;28(4):592–605. doi: 10.1101/gr.227124.117 (PMC5880248; doi:10.1101/gr.227124.117)
Supplement: Supplemental Material [file supp_gr.227124.117_Supplemental_Fig_S4.pdf]

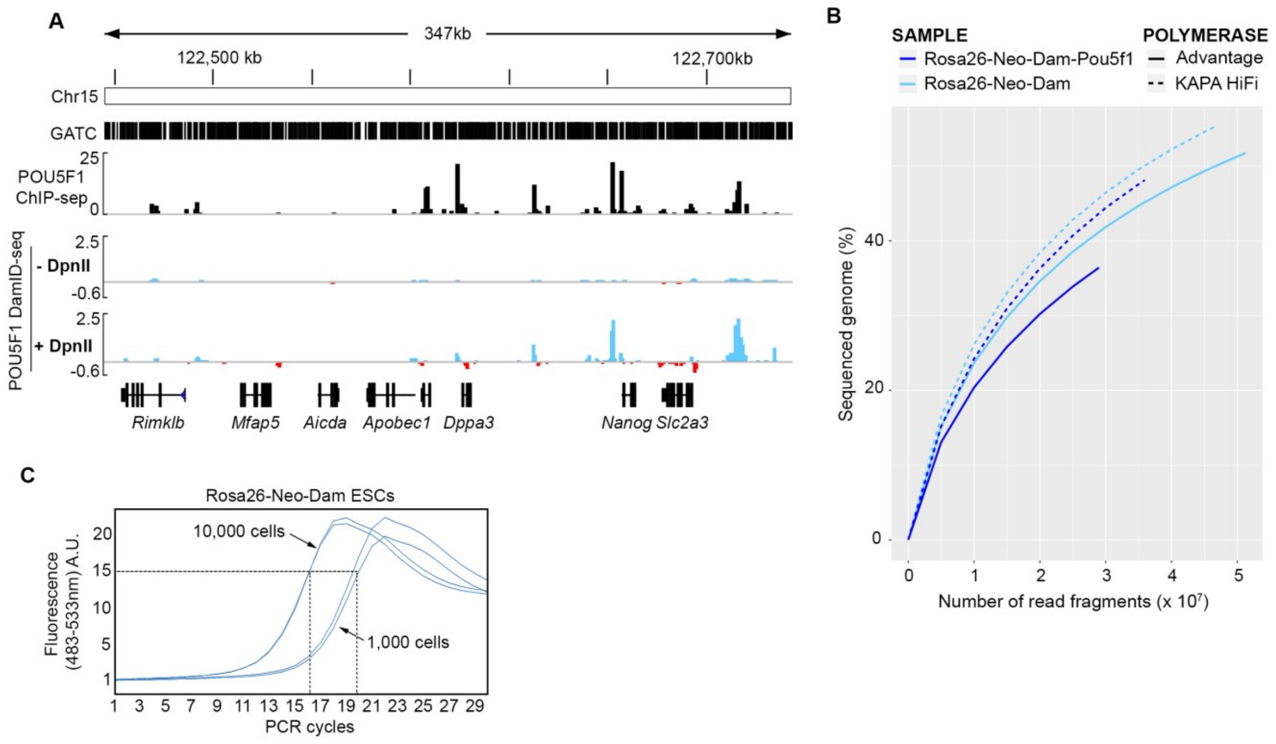

Supplemental Figure S4: Optimization of the DamID-seq protocol. (*A*) A screenshot of POU5F1 DamID-seq tracks generated from 10^4 ESCs with or without the DpnII digestion step (step #4 in Fig. 1B) in comparison with POU5F1 ChIP-seq (Buecker et al. 2014). DamID-seq tracks are represented as the subtraction of DAM-only from DAM-POU5F1 signal (positive signal is in blue and negative signal is in red) (*B*) The percentage of sequenced genome when using Advantage2 Polymerase or KAPA HiFi Polymerase in the PCR amplification step (step #5 in Fig. 1B). (*C*) qPCR analysis to estimate the optimal number of PCR cycles required to amplify each sample. Using this approach, it is possible to determine the number of PCR cycles necessary in order to stop the amplification process in the linear phase (e.g. 16 cycles for 10,000 and 20 cycles for 1,000 Rosa26-Neo-Dam ESCs).
