# Supplementary material for: Mapping transcription factor occupancy using minimal numbers of cells in vitro and in vivo
Source: Genome Res. 2018 Apr;28(4):592–605. doi: 10.1101/gr.227124.117 (PMC5880248; doi:10.1101/gr.227124.117)
Supplement: Supplemental Material [file supp_gr.227124.117_Supplemental_Fig_S5.pdf]

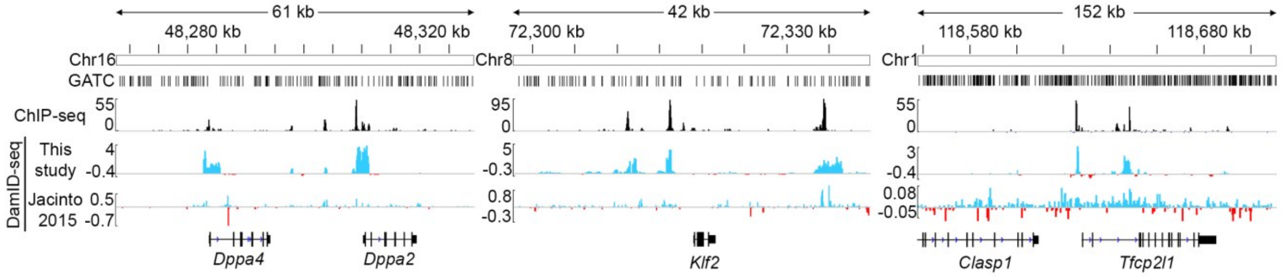

Supplemental Figure S5: Comparison of POU5F1 DamID-seq signal in different studies. POU5F1 binding signal detected by ChIP-seq (Buecker et al. 2014) (top), by DamID-seq in this study with 10^6 cells (middle) and in Jacinto et al. 2015 (bottom) at the 3 different loci. The protocol described in this study improves the signal-to-noise ratio of the DamID-seq signal providing higher agreement with ChIP-seq.
