# Supplementary material for: Mapping transcription factor occupancy using minimal numbers of cells in vitro and in vivo
Source: Genome Res. 2018 Apr;28(4):592–605. doi: 10.1101/gr.227124.117 (PMC5880248; doi:10.1101/gr.227124.117)
Supplement: Supplemental Material [file supp_gr.227124.117_Supplemental_Fig_S7.pdf]

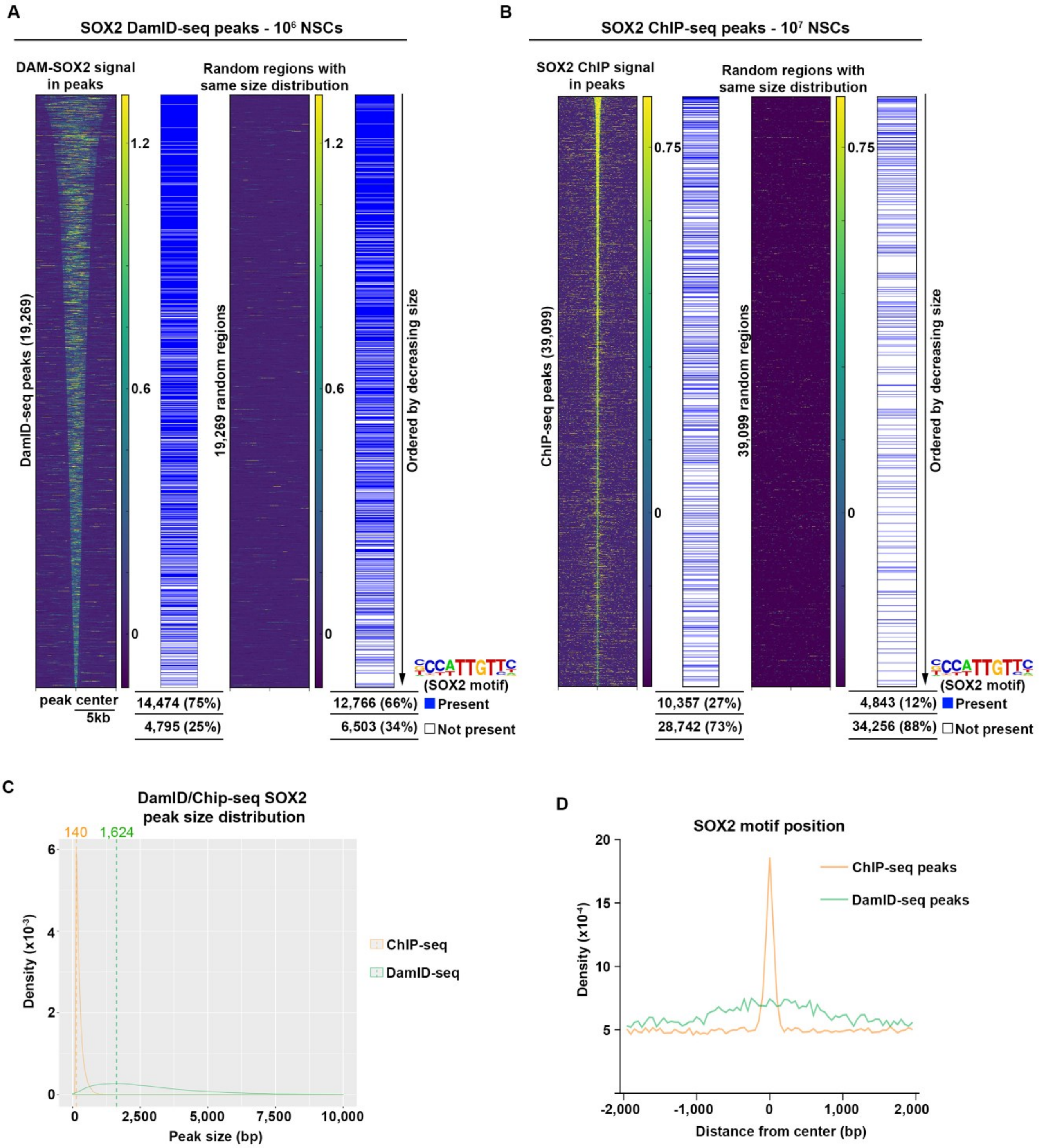

Supplemental Figure S7: SOX2 DamID-seq and ChIP-seq peak features. *(A)* Each row of the left-hand side heatmaps represents SOX2 DamID-seq (A) and ChIP-seq peaks (B) ranked by the size. Heatmaps on the right-hand side represent signal intensity of randomly selected genomic regions with the same sizes. For each peak/randomly selected region, the presence/absence of SOX2 motif is indicated by blue/white lines. (*B*) Density plot of the SOX2 DamID-seq and ChIP-seq peak size. The dashed lines represent the summit value for each group of peaks. (*C*) Distribution of the SOX2 motif within +/-2 Kb from DamID-seq and ChIP-seq peak center.
