# Supplementary material for: Mapping transcription factor occupancy using minimal numbers of cells in vitro and in vivo
Source: Genome Res. 2018 Apr;28(4):592–605. doi: 10.1101/gr.227124.117 (PMC5880248; doi:10.1101/gr.227124.117)
Supplement: Supplemental Material [file supp_gr.227124.117_Supplemental_Fig_S8.pdf]

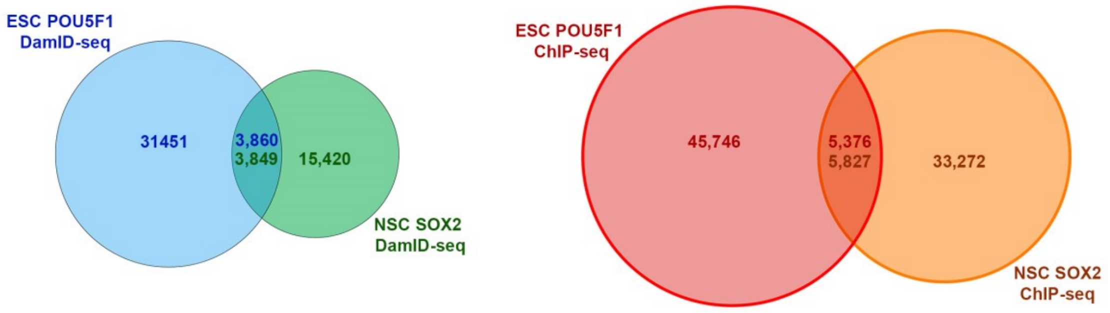

Supplemental Figure S8: Overlaps between 10^6 ESC POU5F1 and 10^6 NSC SOX2 DamID-seq, ESC POU5F1 and NSC SOX2 ChIP-seq. The overlap between POU5F1 binding sites in ESCs and SOX2 binding sites in NSCs is small in either DamID-seq or ChIP-seq.
