# Supplementary material for: Mapping transcription factor occupancy using minimal numbers of cells in vitro and in vivo
Source: Genome Res. 2018 Apr;28(4):592–605. doi: 10.1101/gr.227124.117 (PMC5880248; doi:10.1101/gr.227124.117)
Supplement: Supplemental Material [file supp_gr.227124.117_Supplemental_Fig_S9.pdf]

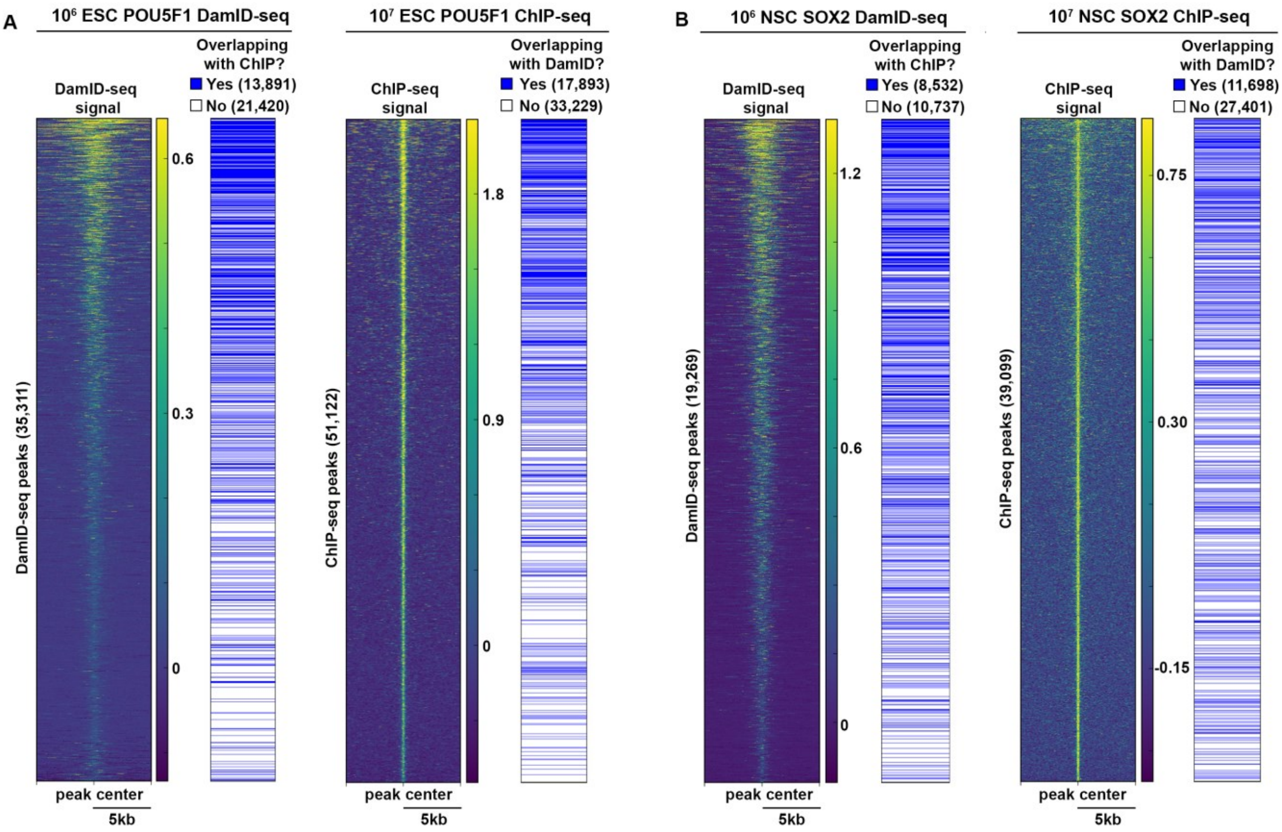

Supplemental Figure S9: Strong peaks in 10^6 ESC DamID-seq and ChIP-seq overlap with each other. *(A, B)* Heatmaps show 10^6 ESC POU5F1 DamID-seq (left) and ChIP-seq (right) peaks (A), 10^6 NSC SOX2 DamID-seq (left) and ChIP-seq (right) peaks (B) ranked by the signal intensity. Blue and white colour next to the heatmaps indicates if each peak has been identified by the other technique.
