# Supplementary material for: Mapping transcription factor occupancy using minimal numbers of cells in vitro and in vivo
Source: Genome Res. 2018 Apr;28(4):592–605. doi: 10.1101/gr.227124.117 (PMC5880248; doi:10.1101/gr.227124.117)
Supplement: Supplemental Material [file supp_gr.227124.117_Supplemental_Fig_S10.pdf]

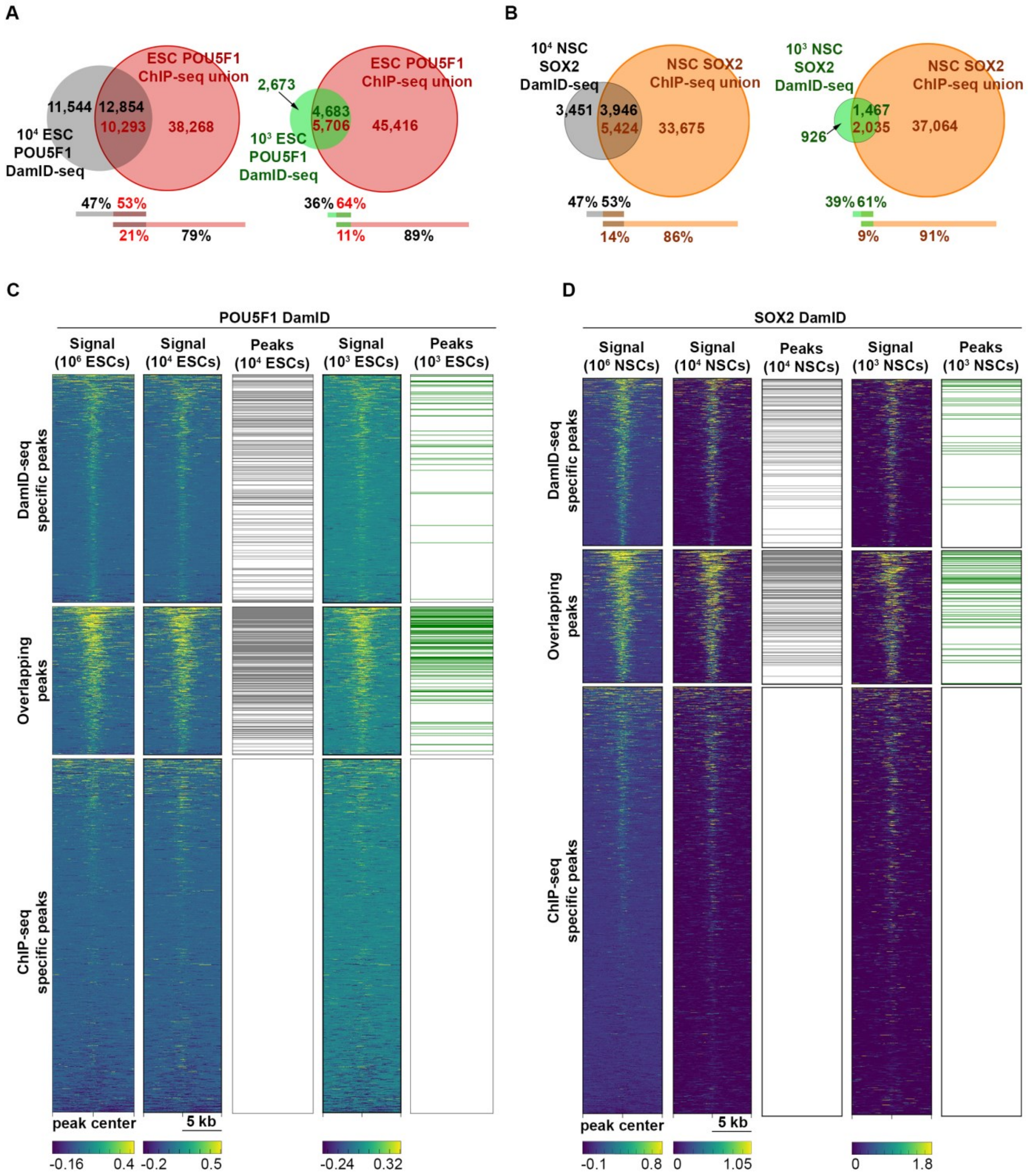

Supplemental Figure S10: DamID-seq peaks from 10^4, 10^3 cells overlap with ChIP-seq peaks (*A, B*) Overlaps between 10^4, 10^3 ESC POU5F1 DamID-seq and ESC POU5F1 ChIP-seq (A), 10^4, 10^3 NSC SOX2 DamID-seq and NSC SOX2 ChIP-seq (B). (*C, D*) Signal intensity in the genomic loci with DamID-seq/ChIP-seq specific and overlapping peaks in the comparison between 10^6 cell DamID-seq and ChIP-seq. POU5F1 DamID-seq signal in $10^6/10^4/10^3$ ESCs (C) and SOX2 DamID-seq signal in $10^6/10^4/10^3$ NSCs are shown. The grey and green lines indicate regions identified as a peak in 10^4 and 10^3 cells, respectively.
