# Supplementary material for: Mapping transcription factor occupancy using minimal numbers of cells in vitro and in vivo
Source: Genome Res. 2018 Apr;28(4):592–605. doi: 10.1101/gr.227124.117 (PMC5880248; doi:10.1101/gr.227124.117)
Supplement: Supplemental Material [file supp_gr.227124.117_Supplemental_Fig_S11.pdf]

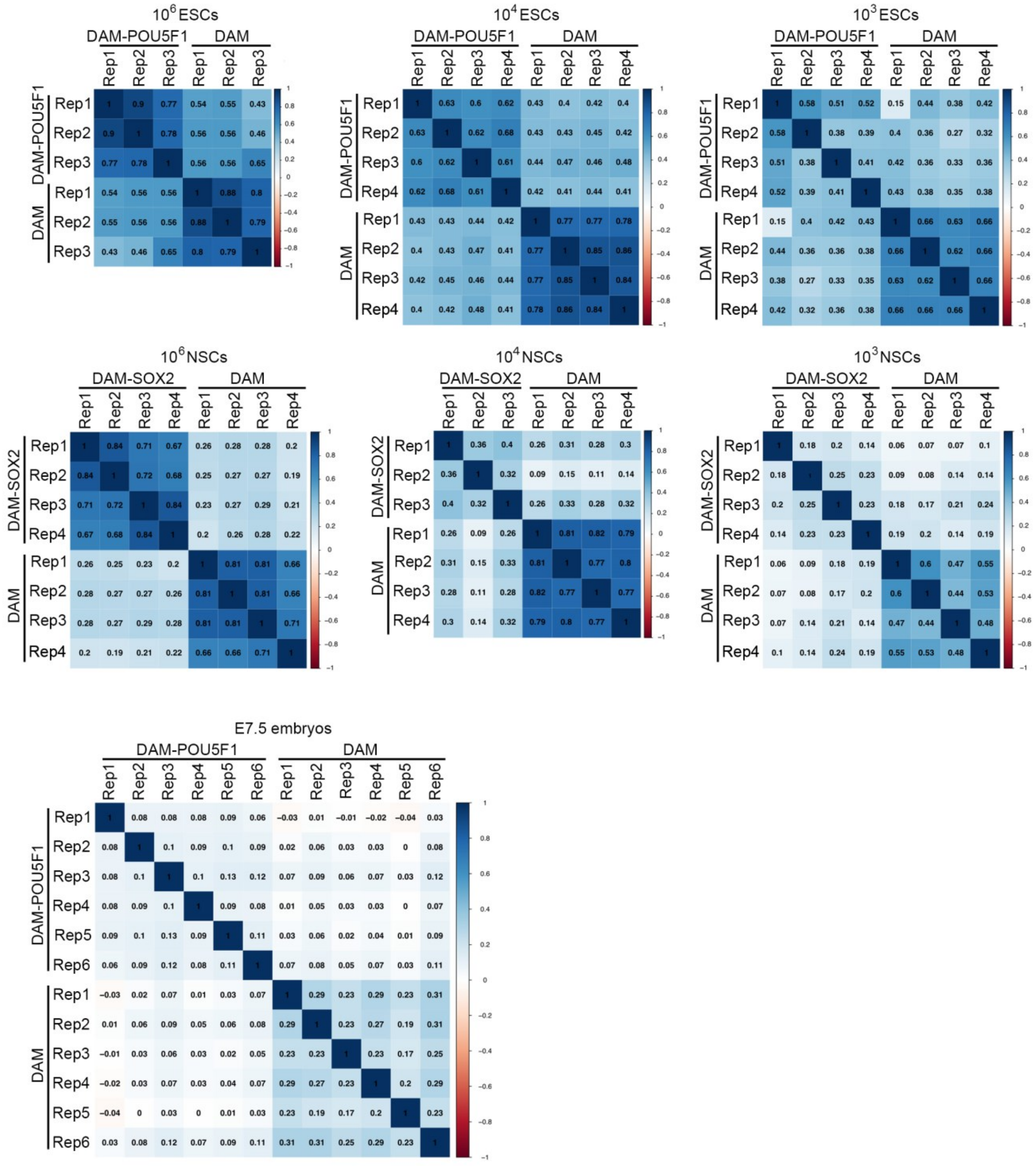

Supplemental Figure S11: Correlation plots of DAM only, DAM-POU5F1 and DAM-SOX2 replicates in 10^6, 10^4, 10^3 ESCs, NSCs, and 7.5 dpc embryos. The heatmaps show the correlation among DAM-only, and DAM-POU5F1 or DAM-SOX2 replicates. Pearson correlation was calculated over each GATC fragment. In general, DAM-only samples show higher correlation than DAM-POI samples. The replicates from lower cell numbers showed lower correlation, and those from 7.5 dpc embryos had the lowest correlation, i.e. the largest variation.
