# Supplementary material for: Mapping transcription factor occupancy using minimal numbers of cells in vitro and in vivo
Source: Genome Res. 2018 Apr;28(4):592–605. doi: 10.1101/gr.227124.117 (PMC5880248; doi:10.1101/gr.227124.117)
Supplement: Supplemental Material [file supp_gr.227124.117_Supplemental_Fig_S12.pdf]

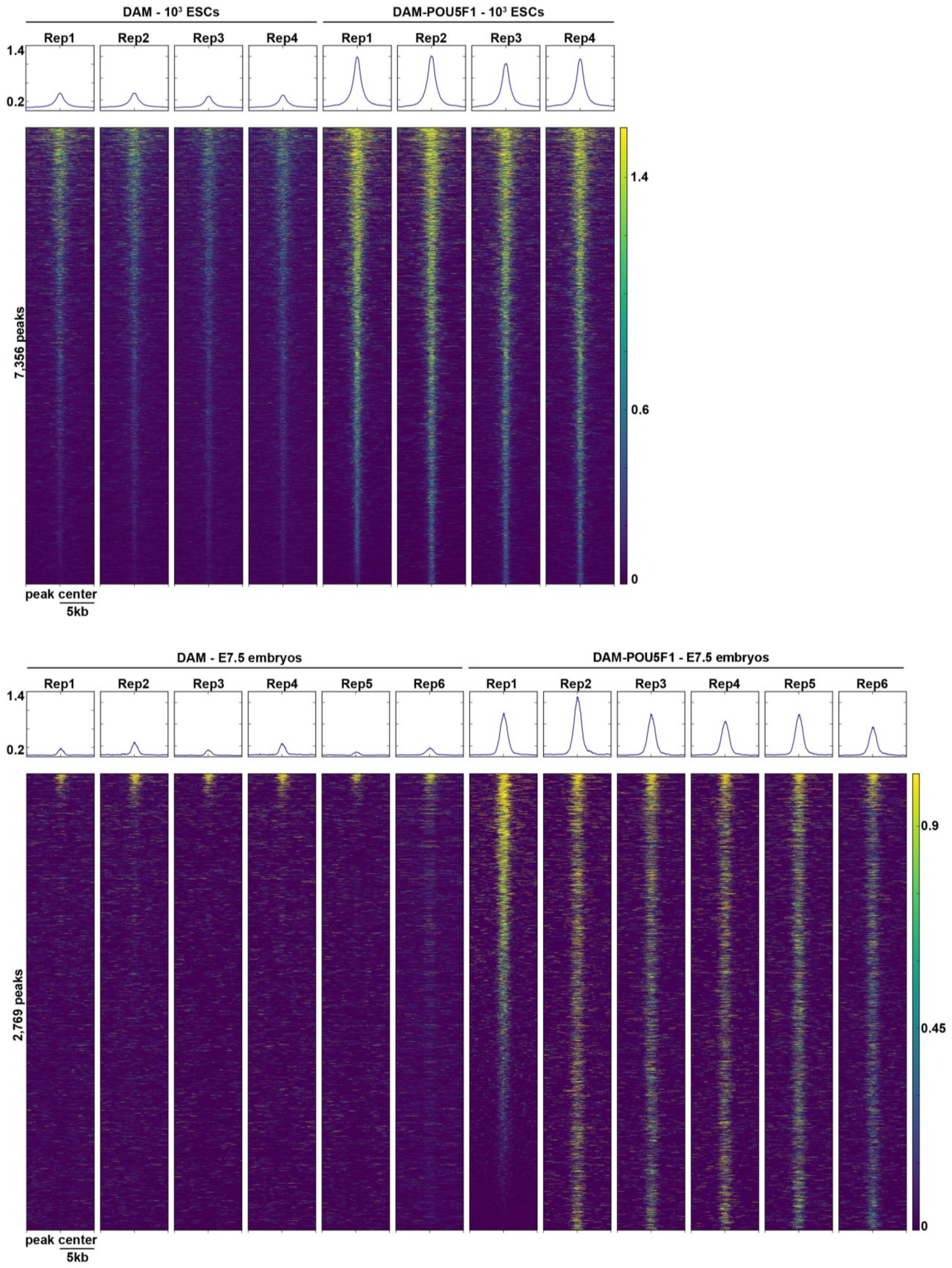

Supplemental Figure S12: DamID-seq signal reproducibility in peak regions. (*A*) DAM-only and DAM-OCT4 signal in the 7,356 peaks identified from 10^3 ESCs and (*B*) and in 2,769 peaks identified from different 7.5 d.p.c. embryos. Despite the overall variability across the genome (Supplemental Fig. S10), DamID-seq signal is highly consistent in the peak regions identified in our peak caller.
