# Supplementary material for: Mapping transcription factor occupancy using minimal numbers of cells in vitro and in vivo
Source: Genome Res. 2018 Apr;28(4):592–605. doi: 10.1101/gr.227124.117 (PMC5880248; doi:10.1101/gr.227124.117)
Supplement: Supplemental Material [file supp_gr.227124.117_Supplemental_Material_S1.tar.gz › analysis.html]

Reference guide for DamID-seq Analysis


# Reference guide for DamID-seq Analysis

#### *James Ashmore*

#### *June 25, 2017*

- Document summary
- Setup environment
- Data pre-processing
- Differential binding analysis

# Document summary

This R Markdown document serves as a reference guide to call peaks from DamID-seq sequencing data. In order to use this document for your own analysis you will need to start with a RangedSummarizedExperiment object. The assay slot in this object should contain your read count matrix. The rows of this matix represent each GATC fragment within the genome. The columns represent the read counts for each sample. The colData slot in this object should contain a data frame with your sample information. One of the columns within this data frame should list which samples are the Dam-POI and Dam-only samples.

# Setup environment

Load required packages:

```
pkgs <- c("csaw", "DESeq2", "edgeR", "genefilter", "limma", "qsmooth", "rtracklayer", "SummarizedExperiment")
libs <- lapply(pkgs, library, character.only = TRUE)
```

Load RangedSummarizedExperiment object (User-input required):

```
rse <- readRDS("ENTER YOUR RSE NAME HERE")
```

Enter column name listing Dam-POI and Dam-only samples (User-input required):

```
covariate <- "ENTER YOUR COLUMN NAME HERE"
```

Calculate median GATC fragment size:

```
fsize <- median(width(rse))
```

# Data pre-processing

Filter low abundance fragments by count size (here we use a default of 10 counts):

```
cutoff <- 10
abundances <- aveLogCPM(assay(rse))
keep <- abundances > aveLogCPM(cutoff, lib.size = mean(colSums(assay(rse))))
rse <- rse[keep, ]
```

Calculate scaling factors:

```
dge <- asDGEList(rse, lib.sizes = colSums(assay(rse)), samples = colData(rse))
normfacs <- normOffsets(rse, lib.sizes = dge$samples$lib.size, type = "scaling")
dge$samples$norm.factors <- normfacs
```

Perform smooth quantile normalization on logCPM values calculated with scaling factors:

```
qsd <- qsmoothData(qsmooth(object = cpm(dge, log = TRUE, prior.count = 1), groupFactor = rse[[covariate]]))
```

# Differential binding analysis

Test for differential binding between Dam-POI and Dam-only samples:

```
design <- model.matrix(~ covariate)
fit <- lmFit(qsd, design)
fit <- eBayes(fit, trend = TRUE, robust = TRUE)
all <- topTable(fit, coef = ncol(design), number = Inf, sort.by = "none")
```

Write test statistics (calculated using limma) for each fragment to file:

```
limmaResults <- data.frame(
    chrom = seqnames(rse),
    chromStart = start(rse) - 1,
    chromEnd = end(rse),
    name = ".",
    score = 0,
    strand = ".",
    logFC = all$logFC,
    AveExpr = all$AveExpr,
    t = all$t,
    P.Value = all$P.Value,
    adj.P.Val = all$adj.P.Val,
    B = all$B
)
write.csv(limmaResults, file = "limmaResults.csv", quote = FALSE, row.names = FALSE)
```

Merge fragments into putative peak regions:

```
merged <- mergeWindows(rowRanges(rse), tol = fsize, max.width = 10000)
results <- data.frame(logFC = all$logFC, AveExpr = all$AveExpr, PValue = all$P.Value)
tabcom <- combineTests(merged$id, results)
tabbest <- getBestTest(merged$id, results)
```

Write putative peak regions (including peak statistics) to file:

```
mergedResults <- data.frame(
    chrom = seqnames(merged$region),
    chromStart = start(merged$region) - 1,
    chromEnd = end(merged$region),
    name = paste0("DamID_Peak_", 1:length(merged$region)),
    score = 0,
    strand = ".",
    signalValue = tabbest$AveExpr,
    logFC = tabbest$logFC,
    pValue = -log10(tabcom$PValue),
    qValue = -log10(tabcom$FDR)
)
write.csv(mergedResults, file = "mergedResults.csv", quote = FALSE, row.names = FALSE)
```

Set qValue (qValue < N) and logFC (logFC > N) peak cutoffs:

```
qValue <- 0.1
logFC <- 0.5
```

Write peaks passing qValue and logFC cutoffs to file:

```
cutoff <- mergedResults$qValue > -log10(qValue) & mergedResults$logFC > logFC
peaks <- mergedResults[cutoff, ]
write.table(peaks, "signifPeaks.broadPeak", quote = FALSE, sep = "\t", row.names = FALSE, col.names = FALSE)
```

Write top differential fragment (used for motif analysis) within each peak region to file:

```
ranges <- rowRanges(rse)[tabbest$best, ]
frags <- data.frame(
    chrom = seqnames(ranges),
    chromStart = start(ranges) - 1,
    chromEnd = end(ranges),
    name = paste0("DamID_Peak_", 1:length(merged$region)),
    score = 0,
    strand = ".",
    signalValue = tabbest$AveExpr,
    logFC = tabbest$logFC,
    pValue = -log10(tabcom$PValue),
    qValue = -log10(tabcom$FDR)
)
cutoff <- mergedResults$qValue > -log10(qValue) & mergedResults$logFC > logFC
frags <- frags[cutoff, ]
write.table(frags, "signifFragments.broadPeak", quote = FALSE, sep = "\t", row.names = FALSE, col.names = FALSE)
```

Print session information:

```
devtools::session_info()
```

```
## Session info -------------------------------------------------------------
```

```
##  setting  value                       
##  version  R version 3.4.0 (2017-04-21)
##  system   x86_64, darwin15.6.0        
##  ui       X11                         
##  language (EN)                        
##  collate  en_GB.UTF-8                 
##  tz       Europe/London               
##  date     2017-06-28
```

```
## Packages -----------------------------------------------------------------
```

```
##  package              * version  date      
##  acepack                1.4.1    2016-10-29
##  annotate               1.54.0   2017-04-25
##  AnnotationDbi          1.38.1   2017-06-01
##  backports              1.1.0    2017-05-22
##  base                 * 3.4.0    2017-04-21
##  base64                 2.0      2016-05-10
##  base64enc              0.1-3    2015-07-28
##  beanplot               1.2      2014-09-19
##  Biobase              * 2.36.2   2017-05-04
##  BiocGenerics         * 0.22.0   2017-04-25
##  BiocParallel         * 1.10.1   2017-05-03
##  biomaRt                2.32.1   2017-06-09
##  Biostrings             2.44.1   2017-06-01
##  bit                    1.1-12   2014-04-09
##  bit64                  0.9-7    2017-05-08
##  bitops                 1.0-6    2013-08-17
##  blob                   1.1.0    2017-06-17
##  bumphunter             1.16.0   2017-04-25
##  checkmate              1.8.2    2016-11-02
##  cluster                2.0.6    2017-03-10
##  codetools              0.2-15   2016-10-05
##  colorspace             1.3-2    2016-12-14
##  compiler               3.4.0    2017-04-21
##  csaw                 * 1.10.0   2017-04-25
##  data.table             1.10.4   2017-02-01
##  datasets             * 3.4.0    2017-04-21
##  DBI                    0.7      2017-06-18
##  DelayedArray         * 0.2.7    2017-06-03
##  DESeq2               * 1.16.1   2017-05-06
##  devtools               1.13.2   2017-06-02
##  digest                 0.6.12   2017-01-27
##  doParallel             1.0.10   2015-10-14
##  doRNG                  1.6.6    2017-04-10
##  edgeR                * 3.18.1   2017-05-06
##  evaluate               0.10.1   2017-06-24
##  foreach                1.4.3    2015-10-13
##  foreign                0.8-69   2017-06-21
##  Formula                1.2-1    2015-04-07
##  genefilter           * 1.58.1   2017-05-06
##  geneplotter            1.54.0   2017-04-25
##  GenomeInfoDb         * 1.12.2   2017-06-09
##  GenomeInfoDbData       0.99.0   2017-05-23
##  GenomicAlignments      1.12.1   2017-05-12
##  GenomicFeatures        1.28.3   2017-06-09
##  GenomicRanges        * 1.28.3   2017-05-25
##  GEOquery               2.42.0   2017-04-25
##  ggplot2                2.2.1    2016-12-30
##  graphics             * 3.4.0    2017-04-21
##  grDevices            * 3.4.0    2017-04-21
##  grid                   3.4.0    2017-04-21
##  gridExtra              2.2.1    2016-02-29
##  gtable                 0.2.0    2016-02-26
##  Hmisc                  4.0-3    2017-05-02
##  htmlTable              1.9      2017-01-26
##  htmltools              0.3.6    2017-04-28
##  htmlwidgets            0.8      2016-11-09
##  httr                   1.2.1    2016-07-03
##  illuminaio             0.18.0   2017-04-25
##  IRanges              * 2.10.2   2017-05-25
##  iterators              1.0.8    2015-10-13
##  knitr                  1.16     2017-05-18
##  lattice                0.20-35  2017-03-25
##  latticeExtra           0.6-28   2016-02-09
##  lazyeval               0.2.0    2016-06-12
##  limma                * 3.32.2   2017-05-02
##  locfit                 1.5-9.1  2013-04-20
##  magrittr               1.5      2014-11-22
##  MASS                   7.3-47   2017-02-26
##  Matrix                 1.2-10   2017-04-28
##  matrixStats          * 0.52.2   2017-04-14
##  mclust                 5.3      2017-05-21
##  memoise                1.1.0    2017-04-21
##  methods              * 3.4.0    2017-04-21
##  minfi                  1.22.1   2017-05-02
##  multtest               2.32.0   2017-04-25
##  munsell                0.4.3    2016-02-13
##  nlme                   3.1-131  2017-02-06
##  nnet                   7.3-12   2016-02-02
##  nor1mix                1.2-2    2016-08-25
##  openssl                0.9.6    2016-12-31
##  parallel             * 3.4.0    2017-04-21
##  pkgmaker               0.22     2014-05-14
##  plyr                   1.8.4    2016-06-08
##  preprocessCore         1.38.1   2017-05-06
##  qsmooth              * 0.0.1    2017-06-28
##  quadprog               1.5-5    2013-04-17
##  R6                     2.2.2    2017-06-17
##  RColorBrewer           1.1-2    2014-12-07
##  Rcpp                   0.12.11  2017-05-22
##  RCurl                  1.95-4.8 2016-03-01
##  registry               0.3      2015-07-08
##  reshape                0.8.6    2016-10-21
##  Rhtslib                1.8.0    2017-04-25
##  rlang                  0.1.1    2017-05-18
##  rmarkdown              1.6      2017-06-15
##  rngtools               1.2.4    2014-03-06
##  rpart                  4.1-11   2017-03-13
##  rprojroot              1.2      2017-01-16
##  Rsamtools              1.28.0   2017-04-25
##  RSQLite                2.0      2017-06-19
##  rtracklayer          * 1.36.3   2017-05-25
##  S4Vectors            * 0.14.3   2017-06-03
##  scales                 0.4.1    2016-11-09
##  siggenes               1.50.0   2017-04-25
##  splines                3.4.0    2017-04-21
##  stats                * 3.4.0    2017-04-21
##  stats4               * 3.4.0    2017-04-21
##  stringi                1.1.5    2017-04-07
##  stringr                1.2.0    2017-02-18
##  SummarizedExperiment * 1.6.3    2017-05-29
##  survival               2.41-3   2017-04-04
##  tibble                 1.3.3    2017-05-28
##  tools                  3.4.0    2017-04-21
##  utils                * 3.4.0    2017-04-21
##  withr                  1.0.2    2016-06-20
##  XML                    3.98-1.9 2017-06-19
##  xtable                 1.8-2    2016-02-05
##  XVector                0.16.0   2017-04-25
##  yaml                   2.1.14   2016-11-12
##  zlibbioc               1.22.0   2017-04-25
##  source                                 
##  CRAN (R 3.4.0)                         
##  Bioconductor                           
##  Bioconductor                           
##  CRAN (R 3.4.0)                         
##  local                                  
##  cran (@2.0)                            
##  CRAN (R 3.4.0)                         
##  cran (@1.2)                            
##  Bioconductor                           
##  Bioconductor                           
##  Bioconductor                           
##  Bioconductor                           
##  Bioconductor                           
##  CRAN (R 3.4.0)                         
##  CRAN (R 3.4.0)                         
##  CRAN (R 3.4.0)                         
##  CRAN (R 3.4.0)                         
##  cran (@1.16.0)                         
##  CRAN (R 3.4.0)                         
##  CRAN (R 3.4.0)                         
##  CRAN (R 3.4.0)                         
##  CRAN (R 3.4.0)                         
##  local                                  
##  Bioconductor                           
##  CRAN (R 3.4.0)                         
##  local                                  
##  CRAN (R 3.4.0)                         
##  Bioconductor                           
##  Bioconductor                           
##  CRAN (R 3.4.0)                         
##  CRAN (R 3.4.0)                         
##  CRAN (R 3.4.0)                         
##  cran (@1.6.6)                          
##  Bioconductor                           
##  CRAN (R 3.4.0)                         
##  CRAN (R 3.4.0)                         
##  CRAN (R 3.4.0)                         
##  CRAN (R 3.4.0)                         
##  Bioconductor                           
##  Bioconductor                           
##  Bioconductor                           
##  Bioconductor                           
##  Bioconductor                           
##  Bioconductor                           
##  Bioconductor                           
##  Bioconductor                           
##  CRAN (R 3.4.0)                         
##  local                                  
##  local                                  
##  local                                  
##  CRAN (R 3.4.0)                         
##  CRAN (R 3.4.0)                         
##  CRAN (R 3.4.0)                         
##  CRAN (R 3.4.0)                         
##  CRAN (R 3.4.0)                         
##  CRAN (R 3.4.0)                         
##  CRAN (R 3.4.0)                         
##  cran (@0.18.0)                         
##  Bioconductor                           
##  CRAN (R 3.4.0)                         
##  CRAN (R 3.4.0)                         
##  CRAN (R 3.4.0)                         
##  CRAN (R 3.4.0)                         
##  CRAN (R 3.4.0)                         
##  Bioconductor                           
##  CRAN (R 3.4.0)                         
##  CRAN (R 3.4.0)                         
##  CRAN (R 3.4.0)                         
##  CRAN (R 3.4.0)                         
##  CRAN (R 3.4.0)                         
##  CRAN (R 3.4.0)                         
##  CRAN (R 3.4.0)                         
##  local                                  
##  cran (@1.22.1)                         
##  cran (@2.32.0)                         
##  CRAN (R 3.4.0)                         
##  CRAN (R 3.4.0)                         
##  CRAN (R 3.4.0)                         
##  cran (@1.2-2)                          
##  CRAN (R 3.4.0)                         
##  local                                  
##  CRAN (R 3.4.0)                         
##  CRAN (R 3.4.0)                         
##  Bioconductor                           
##  Github (stephaniehicks/qsmooth@58f23c4)
##  cran (@1.5-5)                          
##  CRAN (R 3.4.0)                         
##  CRAN (R 3.4.0)                         
##  CRAN (R 3.4.0)                         
##  CRAN (R 3.4.0)                         
##  CRAN (R 3.4.0)                         
##  CRAN (R 3.4.0)                         
##  Bioconductor                           
##  CRAN (R 3.4.0)                         
##  CRAN (R 3.4.0)                         
##  CRAN (R 3.4.0)                         
##  CRAN (R 3.4.0)                         
##  CRAN (R 3.4.0)                         
##  Bioconductor                           
##  CRAN (R 3.4.0)                         
##  Bioconductor                           
##  Bioconductor                           
##  CRAN (R 3.4.0)                         
##  cran (@1.50.0)                         
##  local                                  
##  local                                  
##  local                                  
##  CRAN (R 3.4.0)                         
##  CRAN (R 3.4.0)                         
##  Bioconductor                           
##  CRAN (R 3.4.0)                         
##  CRAN (R 3.4.0)                         
##  local                                  
##  local                                  
##  CRAN (R 3.4.0)                         
##  CRAN (R 3.4.1)                         
##  CRAN (R 3.4.0)                         
##  Bioconductor                           
##  CRAN (R 3.4.0)                         
##  Bioconductor
```
