# Supplementary material for: Mapping transcription factor occupancy using minimal numbers of cells in vitro and in vivo
Source: Genome Res. 2018 Apr;28(4):592–605. doi: 10.1101/gr.227124.117 (PMC5880248; doi:10.1101/gr.227124.117)
Supplement: Supplemental Material [file supp_gr.227124.117_Supplemental_Methods.docx]

Supplemental Methods

***Pou5f1^-/-^* ESC rescue experiment and Alkaline Phosphatase (AP) staining**

Rescue plasmids with *Pou5f1*, *Dam*-Pou5f1*, *Dam** cDNA followed by the *IRES-Puromycin* resistance gene cassette under the CAG promoter were transfected into ZHBTc4 cells (Niwa et al. 2002) using Lipofectamin 3000 (Life Technologies). One day after transfection, cells were replated and cultured in the presence of puromycin (1 μg/ml) and doxycyxline (1 μg/ml) for 12 days. Then, the cells were fixed, washed in distilled water and stained for alkaline phosphatase (AP) expression using a leukocyte alkaline phosphatase kit (Sigma).

**Generation of cell lines via gene targeting and RMCE**

The Rosa26-Neo-Dam, Rosa26-Neo-Dam-Pou5f1, Rosa26-BSD-Dam and Rosa26-Bsd-Dam-Pou5f1 ESC lines were generated via standard gene targeting strategy; the *pROSA26*-*ATTP*-*Neo-Dam-ATTP*, *pROSA26*-*ATTP*-*Neo-Dam-Pou5f1-ATTP, pROSA26*-*ATTP*-*Bsd-Dam-ATTP*, *pROSA26*-*ATTP*-*Bsd-Dam-Pou5f1-ATTP* vectors generated with *pRosa-DEST* (Addgene #21189) were used for the homologous recombination in the *Rosa26* locus of the TNG-MKOS cell line (Chantzoura et al. 2015). The clones with the successful targeting event were identified via southern blot using HindIII- or PacI-digested genomic DNA for the Rosa26 probe or the Neo/Bsd probe, respectively. The primers used to generate the three probes are listed in Supplemental Table S3.

For the φC31-mediated cassette exchange, Rosa26-Bsd-Dam-Pou5f1 (4x10^5^) cells were plated on a gelatinized well of a 6-well dish. 24 hours later, a total of 7 μg of DNA (3.5 μg of vector encoding for φC31 integrase (gift from Prof. Andras Nagy, Mount Sinai Hospital) and 3.5 μg of *ATTB*-*Hyg-Dam-ATTB-pENTR* or *ATTB*-*Hyg-Dam-Pou5f1-ATTB-pENTR*) was used for each transfection using Lipofectamine3000 (Invitrogen). 24 hours after lipofection, cells were replated onto 5 gelatinized 55 cm^2^ dishes and hygromycin (Hyg) selection (150 μg/ml) was started on the next day. Hyg resistant colonies were picked 10 days later, and the successful recombination event was confirmed by genomic PCR using. Primers used for the genomic PCR are listed in Supplemental Table. S3.

**ESC culture**

ES cells were in Glasgow minimum essential medium (GMEM) supplemented with 10% fetal calf serum (FCS), penicillin-streptomycin, 1x non-essential amino acids (Invitrogen), 1 mM sodium pyruvate, 2 mM glutamine, 0.05 mM β-mercaptoethanol (Life Technologies) supplemented with human leukemia inhibitory factor (LIF), 100 U/ml on gelatinized flasks in a 37 °C/5% CO_2_ incubator. For the 2i condition, cells were grown in N2B27 supplemented with 1 μM PD0325901 and 3 μM CHIR99021 (Axon Medchem).

**ESCs to EpiLCs differentiation**

Rosa26-Neo-Dam and Rosa26-Neo-Dam-Pou5f1 ESCs were cultured in serum-free N2B27-based medium (containing DMEM/F12 medium supplemented with N2 combined 1:1 with Neurobasal^®^ medium supplemented with B27; all from Thermo Fisher Scientific), MEK inhibitor (PD0325901, 0.8 μM, Axon Medchem) and GSK3b inhibitor (CHIR99021, 3.3 μM, Axon Medchem) on gelatinized tissue culture dishes (Ying et al. 2008) for at least 5 passages. For EpiLC differentiation, 100,000 cells were replated onto fibronectin-coated (7.5 µg/ml) 22 mm dish in medium serum-free N2B27-based medium supplemented with 1% KOSR, 2 μΜ glutamine (Invitrogen), 0.05 mM β-mercaptoethanol (Thermo Fisher Scientific), 1x non-essential amino acids (Invitrogen), 10 ng/ml FGF2 (R&D), 20 ng/ml ActivinA (Peprotech) for 72 hours before harvesting.

**ESCs to NSCs differentiation**

NSC lines were derived from ESCs as previously described (Pollard et al. 2006). Specifically, *Rosa26-Neo-Dam* and *Rosa26-Neo-Dam-Sox2* ESCs maintained in 2i medium were initially plated on gelatin-coated 6-well plates at 10^6^, 7.5x10^5^ and 5 x10^5^/well in NSC complete medium containing DMEM/F-12 Media, 1:1 Nutrient Mixture (Sigma, D8437), 1X N2 supplement (Thermo Fisher Scientific), 1X B27 supplement (Thermo Fisher Scientific), 8 mM glucose (Sigma, G8644), penicillin-streptomycin, 0.001% BSA (ThermoScientific, 15260037), 0.05 mM β-mercaptoethanol (Thermo Fisher Scientific). Cells were fed daily for 7 days. On day 7, the wells that have more cells with NSC-like morphology were selected, and 1/2 of the cells were seeded into a non-gelatinised flask in NSC complete medium supplemented with 10 ng/ml mouse EGF (Peprotech) and 10 ng/ml human FGF2 (Peprotech). Cells were left to form floating aggregates for 3 days. On day 10, aggregates were harvested by centrifugation of the supernatant at 700 rpm for 3 minutes. Cells were then resuspended in NSC complete media supplemented with EGF, FGF2 and 1 µg/ml Laminin (L2020, Sigma) and seeded onto non-gelatinized T25 flasks. Aggregates were allowed to settle over the course of 10 days, with medium changed every 2-3 days. On day 20, when the flask reached ~50% confluence, half of the cells were passaged into T25 flasks and defined as p1 NSCs. NSCs were maintained in EGF, FGF2, Laminin-supplemented NSC complete medium and passaged every 3-4 days at 1:6/1:8 density. To ensure homogeneity, cells were passaged at least 4 times before performing DamID-seq.

**ESCs to fibroblast differentiation**

Fifty thousand Rosa26-Neo-Dam and Rosa26-Neo-Dam-Pou5f1 ESCs were plated in a gelatin-coated 35 mm dish in ESC medium without LIF, with 1 μM retinoic acid (Sigma) and cultured for 9 days. RNA was extracted from the *in vitro* differentiated fibroblasts and used for qRT-PCR for the expression analysis of mRNA coding Dam protein.

**Dissection of 7.5 dpc epiblasts**

Rosa26-Neo-Dam and Rosa26-Neo-Dam-Pou5f1 chimeric embryos generated by morula aggregation were collected at 7.5 dpc. After removal of the Reichert’s membrane, embryos were imaged to evaluate the presence of *Nanog*-GFP positive cells. The distal part of epiblast was dissected with sharp glass needles in M2 (Sigma, M5910) medium to exclude primordial germ cells. Each epiblast was washed once with PBS, transferred into the Genomic Lysis Buffer provided in the Quick-gDNA™ MicroPrep and processed separately as described in the DamID-seq section for the generation of DamID-seq libraries.

**RNA isolation, cDNA synthesis and quantitative real-time RT-PCR**

RNA was isolated using TRIzol (Life Technologies) according to manufacturer’s instructions. The RNA was then reverse transcribed to cDNA with the Moloney Murine Leukemia Virus Reverse Transcriptase (M-MLV RT, Life Technologies). The qPCR reactions were performed in 384 well plates, in a 10 µl reaction containing cDNA corresponds to 10 ng of starting RNA, 0.5 μM primers and 5 μl LightCycler® 480 SYBR Green I Master Mix (Roche) using LightCycler® 480 (Roche). The primers used for gene expression analyses are listed in Supplemental Table S3.

**CpG methylation data analysis**

*Accession numbers*

CpG methylation data of E14 cells in serum were downloaded from GEO with the GEO DataSet accession number GSE41923.

*Data analysis*

DNA CpG methcounts were lifted over from UCSC mm9 to mm10 assembly of the mouse genome (Church et al. 2009) using CrossMap (Zhao et al. 2014) (version 0.2.2). The ChIP-seq peaks were extended to the same average size of DamID-seq peaks by using the slop utility in BEDTools suite (Quinlan and Hall 2010) and the CpG sites overlapping with DamID-seq, ChIP-seq and common peaks were identified by using the intersect utility in the BEDTools suite (Quinlan and Hall 2010). The average CpG methylation level was calculated and the box-plot was generated using the package ggplot2 in the R statistical environment (version 3.3.2).

**RNA-seq data analysis**

*Accession numbers*

RNA-seq data of ESCs were downloaded from GEO with the GEO DataSet accession number GSE99971. RNA-seq data of *Pou5f1* knockdown in ZHBTc4 ESCs was downloaded from GEO with the BioProject accession number PRJNA347884.

*Sequencing quality*

Sequencing quality was checked using FastQC (version 0.11.5) (Andrews 2014) and contaminant adapters were trimmed using cutadapt (version 1.15) (Martin 2011).

*Transcript quantification*

The trimmed reads were pseudo-aligned to cDNA sequences from UCSC’s knownGene transcriptome (mm10 assembly) using Kallisto (version 0.43.1) with default settings (Bray et al. 2016).

*Differential expression*

Transcript abundances were imported into R and summarised to the gene-level using the tximport workflow (Soneson et al. 2015). Significantly differentially expressed genes (FDR < 0.05) were identified using the standard DESeq2 workflow (Love et al. 2014).

*Peak to gene assignment*
DamID/ChIP-seq specific and overlapping peaks within ±3 kb from TSS were annotated using the annotatePeaks program in the suite HOMER (Heinz et al. 2010) (version 4.8.3). The TPM /logFC/FDR values associated to each gene were calculated and the box-plot and violin plots were generated using the package ggplot2 in the R statistical environment (version 3.3.2).

**DNase-seq data analysis**

*Accession numbers*

DNase-seq hypersensitivity data for E14 mouse embryonic stem cells were downloaded from GEO with the BioSample accession number GSM1014154. DNase-seq data for neural stem cells (NS5 cells) were downloaded from European Nucleotide Archive, accession number ERP004671, sample ERS398430.

*Read coverage visualisation*

Read coverage was lifted over from the UCSC mm9 to mm10 assembly of the mouse genome (Church et al. 2009) using CrossMap (Zhao et al. 2014) (version 0.2.2).

**ChIP-seq data analysis**

*Accession numbers*

POU5F1 ChIP-seq data for E14 mouse embryonic stem cells were downloaded from GEO with the GEO DataSet accession numbers GSE11724, GSE44288 and GSE56138; SOX2 ChIP-seq data for proliferating neural stem cells were downloaded from European Nucleotide Archive, accession numbers PRJEB5253 and PRJNA286988. H3K4me3 and H3K27me3 ChIP-seq data for E14 mouse ESCs were downloaded from GEO with the GEO DataSet accession number GSE23943. H3K27ac and H3K4me1 ChIP-seq data for ESCs were downloaded from GEO with the GEO DataSet accession number GSE56138. H3K4me3 and H3K27me3 ChIP-seq data for neural stem cells were downloaded from GEO with the GEO DataSet accession number GSE46793.

*Sequencing data quality*

Read quality of the downloaded public sequencing data was visualised using charts generated by FastQC (Andrews 2014) (version 0.11.5).

Illumina contaminant adapter sequences were removed using Trim Galore! (Kruger 2012) (version 0.4.3).

*Sequencing data alignment*

The trimmed reads were aligned to the UCSC mm10 assembly of the mouse genome (Church et al. 2009) using BWA MEM (Li and Durbin 2010) (version 0.7.15). For downstream analysis, we focused on just primary mapped reads; unmapped, secondary, and supplemental alignments were filtered using SAMtools (Li et al. 2009) (version 1.4). Reads mapped to uninformative (alternative, unplaced, and mitochondrial contigs) or problematic (ENCODE blacklist) regions (ENCODE Project Consortium 2012) of the genome were also filtered using BEDTools (Quinlan and Hall 2010) (version 2.26.0).

For all ChIP-seq analysis, PCR duplicates were also removed using the MarkDuplicates command from Picard (Broad Institute) (version 2.9.0). Unlike DamID-seq, the probability of fragmenting and sequencing the same exact region is low.

*Peak calling analysis*

ChIP-seq peaks were called using methods developed for ChIP-seq data, rather than using the method we developed for DamID-seq data. It would be unfair to compare technologies using a method designed for one technology, which performs sub-optimally for the other.

For studies without biological replicates (GSE11724 and GSE44288) peak calling was performed by first estimating the fragment size using cross-correlation analysis from the phantompeakqualtools software package (Landt et al. 2012). Peaks were then called using the callpeak command from MACS2 (Liu 2014) (version 2.1.1). The following arguments were used: -g mm --keep-dup all -s <read length> --extsize <estimated fragment size> -q 0.05 -B --SPMR.

For studies with biological replicates (GSE56138 and Sox2 ChIP-seq), peak calling was performed according to the ENCODE (Phase-3) ChIP-seq pipeline (Landt et al. 2012). Briefly, self-pseudoreplicates, pooled replicates, and pooled self-pseudoreplicates were generated for each sample. Fragment size was estimated using cross-correlation analysis from the phantompeakqualtools software package (Landt et al. 2012). Peaks were then called on the self-pseudoreplicates, pooled replicates, and pooled self-pseudoreplicates using the callpeak command from MACS2 (Liu 2014) (version 2.1.1). The following arguments were used: -g mm --keep-dup all -s <read length> --nomodel --shift 0 --extsize <fragment size> -p 0.05 -B --SPMR. High confidence/reproducible peaks were finally identified using an (Li et al. 2011)IDR threshold of 0.05 (version 2.0.3). Peaks identified from the pooled pseudo-replicates dataset were used for the final set of peak calls.

*Read coverage visualisation*

Input-subtracted read coverage was calculated using the bigwigCompare command from deepTools (Ramírez et al. 2016) (version 2.4.3). The bigWig files were generated from the treatment pileup and control lambda bedGraph files from MACS2 using the UCSC bedGraphToBigWig tool (Kent et al. 2010)(version 4).

**Motif enrichment (HOMER) and Gene Ontology (GREAT) analyses**

The motif enrichment analysis was performed with the findMotifsGenome tool of the HOMER (Heinz et al. 2010) suite version 4.8.3. For the motif enrichment, peaks were ranked by signal intensity and the highest 25%, (except 7.5 dpc embryo for which all the 343 peaks), were used as foreground. For the DamID-seq data and DamID-seq/ChIP-seq overlapped peaks, all DAM-only/Dam-POU5F1/SOX2 differentially bound regions (DAM-only enriched, Dam-POU5F1/SOX2enriched including the foreground) were used as background. For the ChIP-seq data, the background was generated by the HOMER software. GREAT analyses (McLean et al. 2010) were performed using GREAT v3.0 using the default “single nearest gene (within 1000kb)” association rule. The highest 25% of each set (ranked by signal intensity) was used as input for each sample; all the 343 peaks identified in the 7.5 dpc embryos were used as input for the GREAT analysis.

**Microarray data analysis of *Pou5f1* conditional KO embryos**

The microarray dataset analysis (GEO accession number GSE46227) was carried out using the statistical programming language R

(R Core Team 2013) (version 3.3.2) and packages from the Bioconductor project (Huber et al. 2015) (version 3.4). Data were normalized and log_2_-transformed by using the limma (Smyth 2004) package (version 3.28.21). The heatmap was generated by using the pheatmap package (version 1.0.8).

**Supplemental References**

Andrews S. 2014. FastQC. A quality control tool for high throughput sequence data. *Babraham Bioinformatics*.

Bray NL, Pimentel H, Melsted P, Pachter L. 2016. Near-optimal probabilistic RNA-seq quantification. *Nature Biotechnology* **34**: 525–527.

Chantzoura E, Skylaki S, Menendez S, Kim S-I, Johnsson A, Linnarsson S, Woltjen K, Chambers I, Kaji K. 2015. Reprogramming Roadblocks Are System Dependent. *Stem Cell Reports* **5**: 350–364.

Church DM, Goodstadt L, Hillier LW, Zody MC, Goldstein S, She X, Bult CJ, Agarwala R, Cherry JL, DiCuccio M, et al. 2009. Lineage-specific biology revealed by a finished genome assembly of the mouse. ed. R.J. Roberts. *PLoS Biology* **7**: e1000112.

ENCODE Project Consortium. 2012. An integrated encyclopedia of DNA elements in the human genome. *Nature* **489**: 57–74.

Heinz S, Benner C, Spann N, Bertolino E, Lin YC, Laslo P, Cheng JX, Murre C, Singh H, Glass CK. 2010. Simple combinations of lineage-determining transcription factors prime cis-regulatory elements required for macrophage and B cell identities. *Molecular Cell* **38**: 576–589.

Huber W, Carey VJ, Gentleman R, Anders S, Carlson M, Carvalho BS, Bravo HC, Davis S, Gatto L, Girke T, et al. 2015. Orchestrating high-throughput genomic analysis with Bioconductor. *Nat Meth* **12**: 115–121.

Kent WJ, Zweig AS, Barber G, Hinrichs AS, Karolchik D. 2010. BigWig and BigBed: enabling browsing of large distributed datasets. *Bioinformatics* **26**: 2204–2207.

Kruger F. 2012. Trim Galore! *Babraham Bioinformatics*.

Landt SG, Marinov GK, Kundaje A, Kheradpour P, Pauli F, Batzoglou S, Bernstein BE, Bickel P, Brown JB, Cayting P, et al. 2012. ChIP-seq guidelines and practices of the ENCODE and modENCODE consortia. *Genome Res* **22**: 1813–1831.

Li H, Durbin R. 2010. Fast and accurate long-read alignment with Burrows-Wheeler transform. *Bioinformatics* **26**: 589–595.

Li H, Handsaker B, Wysoker A, Fennell T, Ruan J, Homer N, Marth G, Abecasis G, Durbin R, 1000 Genome Project Data Processing Subgroup. 2009. The Sequence Alignment/Map format and SAMtools. *Bioinformatics* **25**: 2078–2079.

Li Q, Brown JB, Huang H, Bickel PJ. 2011. Measuring reproducibility of high-throughput experiments. *The Annals of Applied Statistics* **5**: 1752–1779.

Liu T. 2014. Use model-based Analysis of ChIP-Seq (MACS) to analyze short reads generated by sequencing protein-DNA interactions in embryonic stem cells. *Methods Mol Biol* **1150**: 81–95.

Love MI, Huber W, Anders S. 2014. Moderated estimation of fold change and dispersion for RNA-seq data with DESeq2. *Genome Biology* **15**: 550.

Martin M. 2011. Cutadapt Removes Adapter Sequences From High-Throughput Sequencing Reads. *EMBnetjournal* **17**: 10–12.

McLean CY, Bristor D, Hiller M, Clarke SL, Schaar BT, Lowe CB, Wenger AM, Bejerano G. 2010. GREAT improves functional interpretation of cis-regulatory regions. *Nature Biotechnology* **28**: nbt.1630–9.

Niwa H, Masui S, Chambers I, Smith AG, Miyazaki J-I. 2002. Phenotypic complementation establishes requirements for specific POU domain and generic transactivation function of Oct-3/4 in embryonic stem cells. *Molecular and Cellular Biology* **22**: 1526–1536.

Pollard SM, Benchoua A, Lowell S. 2006. Neural stem cells, neurons, and glia. *Meth Enzymol* **418**: 151–169.

Quinlan AR, Hall IM. 2010. BEDTools: a flexible suite of utilities for comparing genomic features. *Bioinformatics* **26**: 841–842.

R Core Team. 2013. R: A language and environment for statistical computing.

Ramírez F, Ryan DP, Grüning B, Bhardwaj V, Kilpert F, Richter AS, Heyne S, Dündar F, Manke T. 2016. deepTools2: a next generation web server for deep-sequencing data analysis. *Nucleic Acids Research* **44**: W160–5.

Smyth GK. 2004. Linear models and empirical bayes methods for assessing differential expression in microarray experiments. *Stat Appl Genet Mol Biol* **3**: Article3–25.

Soneson C, Love MI, Robinson MD. 2015. Differential analyses for RNA-seq: transcript-level estimates improve gene-level inferences. *F1000Res* **4**: 1521.

Ying Q-L, Wray J, Nichols J, Batlle-Morera L, Doble B, Woodgett J, Cohen P, Smith A. 2008. The ground state of embryonic stem cell self-renewal. *Nature* **453**: 519–523.

Zhao H, Sun Z, Wang J, Huang H, Kocher J-P, Wang L. 2014. CrossMap: a versatile tool for coordinate conversion between genome assemblies. *Bioinformatics* **30**: 1006–1007.
