# Supplementary material for: Mapping transcription factor occupancy using minimal numbers of cells in vitro and in vivo
Source: Genome Res. 2018 Apr;28(4):592–605. doi: 10.1101/gr.227124.117 (PMC5880248; doi:10.1101/gr.227124.117)
Supplement: Supplemental Material [file supp_gr.227124.117_Supplemental_Table_S1.docx]

| **Reference** | **Year** | **Transcription factor of interest** | **Number of cells** | **Cell type** | **Expression system** | **Technology** | **Further comments** |
| --- | --- | --- | --- | --- | --- | --- | --- |
| Vogel et al.  Nature Protocol | 2007 | N/A | 2.5 μg of purified DNA  (>10^6^ cells) | MCF7, Tig3ET, HeLa, 293T, U2OS, BJ, PALO4AD, Ramos Burkitt’s lymphoma cells, MEFs | Viral transduction | DamID-chip | Original protocol for DamID in mammalian cells |
| Lie-A-Ling et al.  Blood | 2014 | *Runx1* | as in Vogel et al. | Mouse ESC-derived haemogenic endothelium (HE) cells | Dam/Dam-Runx1 targeted to *Ainv18* locus | DamID-seq | ABI Solid Sequencing, 9268 peaks identified |
| Artegiani et al.  EMBO J | 2015 | *Tox* | as in Vogel et al. | Neuro2a and HEK293 | Viral transduction | DamID-seq | Illumina HiSeq, Tox  binding not detected in Neuro2a cells |
| Bouveret et al.  eLife | 2015 | *NKX2-5, Elk1, Elk4* | as in Vogel et al. | HL-1 | Viral transduction | DamID-chip | Affymetrix mouse 1.0R promoter microarrays, ~1217 peaks identified |
| Jacinto et al.  Genes & Dev | 2015 | *Oct4* | as in Vogel et al. | Mouse ESCs | Viral transduction | DamID-seq | DpnII digestion not performed before PCR amplificaiton, Illumina HiSeq, numbers of identified peaks not described |
| Thambyrajah et al.  Nature Cell Biol | 2015 | *Gfi1/Gfi1B* | as in Vogel et al. | Mouse ESC-derived haemogenic endothelium (HE) cells | Dam/Dam-Gfi1/Dam-Gfi1b targeted to *Ainv18* locus | DamID-seq | Illumina HiSeq, numbers of identified peaks not described |
